# Evaluating the MedMira Multiplo® Complete Syphilis (TP/nTP) antibody test in a sexually transmitted infection clinic in Ottawa, Canada: increased rapid diagnosis and improved antibiotic stewardship

**DOI:** 10.1186/s12879-025-12263-w

**Published:** 2025-12-08

**Authors:** Patrick O’Byrne, Riley Dillabough, Kathleen Whyte, Lauren Orser, Vanessa Tran, Venkata R. Duvvuri, Raymond Tsang

**Affiliations:** 1 School of Nursing, University of Ottawa, 125 University Private, Ottawa, Ontario K1N 1A2 Canada; 2 Ottawa Public Health, Sexual Health Clinic, 179 Clarence Street, Ottawa, Ontario K1N 5P7 Canada; 3 Laboratory for Syphilis Diagnostics, National Microbiology Laboratory Branch, Public Health Agency of Canada, Winnipeg, Manitoba Canada; 4 Public Health Ontario, 661 University Avenue, Suite 1701, Toronto, ON M5G 1M1 Canada; 5 Department of Laboratory Medicine and Pathobiology, University of Toronto, Temerty Faculty of Medicine, 1 King’s College Cir, Toronto, ON M5S 3K3 Canada

**Keywords:** Point-of-care testing, Syphilis diagnosis, Syphilis treatment, Antimicrobial stewardship

## Abstract

**Background:**

Syphilis now affects every population and serology is the mainstay of diagnosis. The issue is that serology has a turnaround time of several days. One solution is point-of-care tests (POCTs), which can provide results in minutes. We consequently evaluated the MedMira Multiplo® Complete Syphilis Test in an STI clinic in Ottawa, Canada.

**Methods:**

Anyone 16 + years old who consented and was undergoing syphilis testing at our clinic was eligible. Those who enrolled completed the POCT and saw a clinician to review their result. We calculated sensitivities and specificities for the POCT, compared to serology and diagnosis.

**Results:**

From August 2024 to May 2025, we performed 622 syphilis POCTs on 600 participants. Compared to serology when chemiluminescent microparticle immunoassay (CMIA) and *Treponema pallidum* particle agglutination (TP.PA) tests were reactive, the POCT treponemal (TP) test had a sensitivity of 90.1% and specificity of 97.9%. Compared to any dilution of rapid plasma reagin (RPR), the POCT non-treponemal (nTP) test had a sensitivity of 82.5% and specificity of 99.1%. When we stratified POCT nTP results based on RPR titers, the POCT nTP had a sensitivity of 94.1% for RPR dilutions ≥ 1:8. Compared to serology, the POCT identified 91.4% of new syphilis infections and 97% of infectious syphilis.

**Conclusions:**

POCTs informed clinical syphilis management. While most research has focused on how POCTs can facilitate treatment, in our study, there was a second major utility: to withhold antibiotics when recommended as empiric treatment but when the patient does not have active syphilis. Future research on syphilis POCTs should focus on their abilities to rule in and rule out infections.

**Trial registration:**

NCT06586905 (Registered Sept 4, 2024).

**Supplementary information:**

The online version contains supplementary material available at 10.1186/s12879-025-12263-w.

## Introduction

Syphilis is a sexually transmitted infection (STI) that progresses from a localized lesion at the site of inoculation (primary stage) to a disseminated infection with systemic symptoms (secondary stage) to a latent infection (early latent if < 12 months from acquisition and late latent if > 12 months); in certain persons, syphilis can reactivate years later and cause destruction of any tissue (tertiary stage) [1]. Primary, secondary, and early latent syphilis can be transmitted sexually at rates of 30–60% [1], and vertically at rates of 40–100% [2]. Late latent syphilis is non-infectious for sexual transmission, but can result in vertical transmission at rates of 10% [1].

Adding to this clinical complexity is that syphilis rates have increased over the last 20 years, with the epidemiologic profile having changed [2–5]. In Canada, the United States, and the United Kingdom, while men who have sex with men (MSM) accounted for most diagnoses in the 2010s, by 2025, there were increases in syphilis in heterosexual men and women, which has caused a resurgence of congenital syphilis – an infection that was heretofore rare in Western countries [2–5]. In summary, the current context of syphilis is thus one wherein (1) it often presents asymptomatically and when symptomatic is difficult to recognize, and (2) with changes in the epidemiology of syphilis, it now affects more people and members of virtually every population [1, 2, 6].

Considering this background, clinical guidelines for syphilis from Canada and the United States (and most other jurisdictions) recommend that practitioners screen all sexually active persons with risk factors and consider empirically treating those with risk factors and suggestive symptoms [6, 7]. Such screening occurs via serology, with, in our context of Ontario, Canada, a 3–7 day turnaround time [8]. Asymptomatic patients who test positive must then return to clinic for treatment, which can result in onward syphilis transmission if the patient had new sexual partners between when they underwent testing and received treatment. In contrast, empirically treating symptomatic patients may overuse antibiotics, making the clinical management of syphilis a balancing act between prompt treatment of true infections (to eliminate infection and minimize onward transmission), and antibiotic stewardship (to minimize antibiotic resistance).

Point-of-care tests (POCTs) [9, 10] are one possible aid to clinical decision-making for syphilis management, as these devices can help identify persons needing treatment (when the POCT is positive in the context of risk factors for syphilis); alternatively, POCTs can support decisions to withhold treatment (when the POCT is negative in symptomatic patients). To date, however, the utility of syphilis POCTs in Canada has been limited by device performance – with, at the time of our study, the single device approved in Canada only being able to detect treponemal (TP) antibodies (i.e., the INSTI® multiplex) [11]. That is, when we completed this study, no device was licensed in Canada that could detect TP and non-treponemal (nTP) antibodies. The outcome was that it remained difficult to clinically interpret results from this TP-only device involving persons with treated infections. Internationally, however, research has shown that dual TP/nTP tests can differentiate untreated from treated historical syphilis infections with good performance [12–14]. These devices thus warrant further exploration.

To add to this knowledge base on dual TP/nTP POCTs, in our STI clinic in Ottawa, Canada, we completed the first clinical trial involving a device that was unlicensed at the time of our study: the MedMira Multiplo® Complete Syphilis (TP/nTP) Antibody Test [15]. In undertaking this study, we sought to answer the following two-part question: When frontline nurses in an STI clinic in Canada were trained to incorporate the MedMira Multiplo® Complete Syphilis (TP/nTP) POCT into their routine clinical practice, (1) What was the accuracy of this device compared to conventional serology? and (2) What were the patient outcomes associated with clinical implementation of this device? Real-world data from intended-use settings (i.e., STI clinics in larger urban centers) are crucial to understanding the utility of POCTs [16] and to inform considerations about if and how they should be licensed for use.

## Methods

### Design & setting

This observational cross-sectional study occurred in Ottawa, Canada, which is the fourth largest metropolitan area in Canada, with a population of ~ 1.5 million people. Ottawa, furthermore, has had ~ 200-325 reported diagnoses of syphilis per year since 2016. The study site for this research was a public health STI clinic, which, from 2016–2025, accounted for an average of 30% of all diagnoses of infectious syphilis per year in Ottawa.

For background, our clinic is a general STI clinic that is accessible to anyone with risk factors for STI or HIV acquisition. All services are free. We offer point-of-care testing for HIV, serologic testing for HIV, syphilis, hepatitis A and B and C, and urine and oral and rectal swab testing for gonorrhea and chlamydia. Based on clinical indication, we also test for herpes simplex virus, mpox, bacterial vaginosis, yeast, and trichomoniasis. All testing is performed through Public Health Ontario’s laboratory. We also provide immunizations against HPV, mpox, and hepatitis A and B. Our clinic is staffed by a mixture of physicians, nurse practitioners, and registered nurses, who work in full and part-time capacities.

All nurses who worked in our clinic providing frontline patient care (n = 8) were trained to use the device. Before study initiation, all nurses completed validation testing using stored samples to perform the test. These nurses had to read the results correctly in a blind evaluation with the study coordinator (who was trained directly by the manufacturer). All study nurses had to complete a mid-study recertification with a second blinded evaluation for the device as well.

### Eligibility & participation

The study was open to anyone who presented to our clinic for testing, was ≥ 16 years of age, and could review the consent form. All eligible persons were offered enrollment at check-in to our clinic. Participation included seeing a study nurse to review and sign the consent form, have serology drawn for conventional syphilis testing, and complete the syphilis POCT. Consent was obtained to complete the POCT and to extract data from the participant’s medical record for analysis. The POCT was performed according to manufacturer’s directions using fingerstick blood with the patient present. Upon completion of the POCT, the study nurse presented the POCT results to the patient and clinician who completed the clinical visit, at which time shared decision-making based on history, examination, and the POCT result determined next steps: administer treatment immediately or defer treatment pending serology results. At this time, the clinician who saw the patient would perform all other indicated testing based on the patient’s history and examination.

### Sample size calculation

We used procedures described by Banoo et al. [17] to calculate the required sample size based on a desired POCT sensitivity of 90% and minimum sensitivity of 80%. The minimum sample size (N) of participants with positive syphilis antibodies (detection of TP antibodies regardless of nTP activity) required to demonstrate, with statistical confidence, that the POCT meets the minimum sensitivity threshold of 80% was calculated using the following formula:

$$\rm{N} = {\left( {{Z_1}\_\alpha /_2 + {Z_1}\_\beta } \right)^2} \times \left[ {\rm{p}\left( {1-\rm{p}} \right)/{{\left( {\rm{p}-{\rm{p}_0}} \right)}^2}} \right]$$

Where Z₁₋⍺/₂ is the Z-score corresponding to the desired confidence level at 95%, or 1.96, Z₁₋β is the Z-score corresponding to the desired statistical power at 90% or 1.28; p is the desired POCT sensitivity, 90% or 0.90; and p_0_ is the minimum acceptable sensitivity, 80% or 0.80.

$$\begin{aligned}{\mathrm{N}} = &\:{\left( {{\mathrm{1}}{\text{.96 + 1}}{\mathrm{.28}}} \right)^{\mathrm{2}}}\\&\times\left[ {{\mathrm{0}}{\text{.90 }}\left( {{\text{1 -- 0}}{\mathrm{.90}}} \right){\mathrm{/}}{{\left( {{\mathrm{0}}{\text{.90 -- 0}}{\mathrm{.80}}} \right)}^{\mathrm{2}}}} \right]\end{aligned}$$

$${\text{N = }}{\left( {{\mathrm{3}}{\mathrm{.24}}} \right)^{\mathrm{2}}}{\text{x }}\left[ {{\mathrm{0}}{\text{.90 }}\left( {{\mathrm{0}}{\mathrm{.10}}} \right){\mathrm{/}}{{\left( {{\mathrm{0}}{\mathrm{.1}}} \right)}^{\mathrm{2}}}} \right]$$

$${\text{N = 10}}{\text{.4976 x }}\left[ {{\mathrm{0}}{\text{.09 / 0}}{\mathrm{.01}}} \right]$$

$${\text{N = 95}}$$

To detect with 95% confidence that the POCT had a sensitivity of at least 80%, our sample size needed to contain at least 95 participants who tested positive for syphilis antibodies.

To minimize false positives, we set a desired specificity of 95% and a minimum acceptable specificity of 90%. The minimum sample size (n) of participants without the diagnosis of acute or infectious syphilis required for the study was calculated by the formula:

$$\rm{n} = {\left( {{Z_1}\_\alpha /_2 + {Z_1}\_\beta } \right)^2} \times \left[ {p\left( {1-p} \right)/{{\left( {p-{p_0}} \right)}^2}} \right]$$

As above, where Z₁₋⍺/₂ is the Z-score for the desired confidence level at 95%, or 1.96, Z₁₋β is the Z-score for the desired power at 90% or 1.28; q is the desired POCT specificity, 95% or 0.95; and p_0_ is the minimum acceptable specificity, 90% or 0.90.

$$\begin{aligned}{\mathrm{N}} =\: &{\left( {{\mathrm{1}}{\text{.96 + 1}}{\mathrm{.28}}} \right)^{\mathrm{2}}}\\&\times\left[ {{\mathrm{0}}{\text{.95 }}\left( {{\text{1 -- 0}}{\mathrm{.95}}} \right){\mathrm{/}}{{\left( {{\mathrm{0}}{\text{.95 -- 0}}{\mathrm{.90}}} \right)}^{\mathrm{2}}}} \right]\end{aligned}$$

$${\text{N = }}{\left( {{\mathrm{3}}{\mathrm{.24}}} \right)^{\mathrm{2}}}{\text{x }}\left[ {{\mathrm{0}}{\text{.95 }}\left( {{\mathrm{0}}{\mathrm{.05}}} \right){\mathrm{/}}{{\left( {{\mathrm{0}}{\mathrm{.05}}} \right)}^{\mathrm{2}}}} \right]$$

$${\text{N = 10}}{\text{.4976 x }}\left[ {{\mathrm{0}}{\mathrm{.0475/0}}{\mathrm{.0025}}} \right]$$

$${\text{N = 199}}$$

To detect with 95% confidence that the POCT had a minimum specificity of 90%, we needed at least 199 participants who tested negative for syphilis antibodies.

Using these formulae, we determined that, with a syphilis TP antibody positivity rate of ~ 16% by serology in our clinic, we needed to recruit 600 participants to enrol 95 persons who would test positive for syphilis antibodies and at least 199 who would test negative. Because the POCT we evaluated was unlicensed at the time of the study, we obtained approval from Health Canada to perform up to 800 tests on 600 unique participants. Recruitment occurred until we recruited the 600^th^ person, on a first-come-first-serve basis to produce a convenience sample.

### Testing

Locally, syphilis screening occurs by serology and follows the reverse algorithm. Testing is performed using the Abbott Alinity system [18] with a qualitative chemiluminescent microparticle immunoassay (CMIA). Reflex testing involves a rapid plasma reagin (RPR) test using the automated Gold Standard Diagnostics RPR Test System [19] and Pulse Scientific RPR Carbon Antigen Kit [20], if requiring manual dilution, and a *Treponema pallidum* particle agglutination (TP.PA) test using the Serodia TP–PA.

The first step in this testing flow [8] is the qualitative CMIA, which detects TP-specific IgG and IgM antibodies, without distinguishing which is present. No further testing occurs when the CMIA is non-reactive. When the CMIA is reactive, reflex RPR testing occurs. The RPR detects nTP antibodies to cardiolipin-lecithin-cholesterol and yields titers (1:1, 1:2, 1:4, etc.). No further testing occurs if the RPR is reactive. If the RPR is non-reactive, the TP.PA test is performed, but only if there is no reactive TP.PA result on file with the laboratory. The TP.PA is a second TP antibody test for IgG and IgM (without distinguishing between which is present) and can confirm the CMIA result. Results are reported as follows: CMIA non-reactive; CMIA reactive with an RPR titer; or CMIA reactive, RPR non-reactive, and TP.PA as reactive or previous reactive. The CMIA is ~ 75% sensitive for primary infections and effectively 100% sensitive for all other stages, while the RPR is 60–90% sensitive for primary infections, virtually 100% sensitive for secondary infections, and ~ 75% sensitive for late latent and tertiary infections [1].

The POCT we evaluated was the MedMira Multiplo® Complete Syphilis (TP/nTP) Antibody Test [15], which is “a manually performed, visually interpreted, rapid vertical flow immunoassay” that detects TP and nTP antibodies. The MedMira Multiplo® Complete Syphilis (TP/nTP) Antibody Test [15] uses the same recombinant antigens for its TP component as the Abbott CMIA test (Alinity System). Performing the test requires 30 µL of fingerstick blood, which is mixed with a lysing agent and poured into the test device. Next, the test cap is placed over the device, and a second buffer agent is poured into the device through the test cap. The test cap is removed, and results are interpreted once the liquid is fully absorbed. The time taken to complete this test is 2–3 min.

Three qualitative test results can appear: the control line, the TP dot, and the nTP dot. Test results can be invalid (no control line, with or without other dots), non-reactive (control line present and TP/nTP dots not visible), or reactive (control line, TP and/or nTP dots visible). Using 10 serum/plasma specimens in a laboratory, the manufacturer found the device was 100% sensitive for TP/nTP antibodies when serology results were RPR ≥ 1:8 [15]. To date, there are no published clinical trials using this device (making our results the first using this POCT).

### Syphilis staging

The clinical management of syphilis is not based on laboratory results alone. Instead, it is the culmination of decision making based on patient history, examination, and test results to stage patients according to the natural history of syphilis: primary, secondary, early or late latent, or tertiary syphilis. (See Box 1 for syphilis case definitions, per the Public Health Agency of Canada [21]). We have previously published an algorithm detailing this diagnostic process, based on the PHAC case definitions for the stages of syphilis [22]. (See staging algorithm in Supplemental.) This algorithm guided how we managed serologic syphilis lab results for this study.

**Box 1:** Syphilis case definitions (per the Public Health Agency of Canada)

Stage	Requirements
Primary	*• Treponema pallidum* identification by direct method (e.g., PCR, DFA, dark-field), or *•* In persons without historical syphilis infection, reactive TP serology with the presence of primary syphilis lesions (i.e., chancre), or *•* In persons with historical syphilis infection, a ≥ 4-fold increase in nTP titer from a prior nTP titer with the presence of primary syphilis lesions (i.e., chancre)
Secondary	*• Treponema pallidum* identification by direct method (e.g., PCR, DFA, dark-field) and reactive TP and nTP serology, or *•* In persons with historical syphilis infection, a ≥ 4-fold increase in nTP titer from a prior nTP titer with the presence of secondary syphilis lesions (i.e., mucus lesions, rash, generalized lymphadenopathy, flu-like symptoms)
Early latent	*•* Reactive TP and/or nTP serology in an asymptomatic person who, in the previous 12 months, had one of the following: ◦Non-reactive TP serology ◦Unequivocal symptoms of primary or secondary syphilis ◦Exposure to a partner with infectious syphilis (i.e., primary, secondary, or early latent syphilis)
Late latent	• Reactive TP and/or nTP serology in an asymptomatic person who does not meet the criteria for early latent syphilis

Abbreviations: DFA = direct fluorescence antibody; PCR = polymerase chain reaction; nTP = non-treponemal; TP = treponemal

### Data collection

We extracted data for this study from participants’ medical records, and included age, sex, presence of symptoms (including which, if present), prior syphilis history, POCT results, treatment decision at the point-of-care, syphilis serology results, and final syphilis staging [21].

### Data analysis

We calculated sensitivities and specificities for the POCT TP and nTP, compared to CMIA and RPR, and compared to clinical diagnosis. Predictive values were calculated as the total number of true positives or negatives divided by the combined total of true positives or negatives plus false positives or negatives.

More specifically, sensitivity metrics for the POCT to detect syphilis antibodies were defined as the percentage of tests with laboratory confirmed antibodies identified as positive by the POCT. This was calculated by taking the number of tests that were positive by POCT (TP and then nTP), divided by the number of tests that were positive by conventional TP and nTP tests, respectively; and multiplied by 100%. Specificity of the POCT to detect syphilis antibodies was defined as the percentage of tests without laboratory confirmed antibodies identified as negative by the POCT. This was calculated by taking the number of tests that were negative by POCT (TP and/or nTP) divided by tests that were negative by conventional TP and nTP tests, respectively; and multiplied by 100%. The false positive rate for the POCT was calculated by taking the number of tests that were positive by POCT (TP and then nTP), divided by the number of tests that were negative by conventional TP and nTP serology, respectively; and multiplied by 100%. The false negative rate for the POCT was defined as the percentage of tests that were negative by POCT but positive by conventional serology (for both TP and nTP). This was calculated by dividing the number of negative POCT results (TP and nTP) with total number of tests that were positive by conventional serology; and multiplied by 100%. Positive predictive values were calculated as the total number of true positives divided by the combined total of true positive plus false positive results. Negative predictive values were calculated similarly but using the negative results. In both cases, disease prevalence was calculated as the seroprevalence of antibodies for that test.

Another set of sensitivities, specificities, false positive rates, and false negative rates were determined against the STI clinician’s final diagnosis on the participants’ syphilis status (active, past, or no syphilis infection) using clinical presentation, conventional laboratory tests for syphilis and a direct syphilis detection test, i.e., the direct fluorescence antibody (DFA) tests. Definitions for these infections followed those detailed by the Public Health Agency of Canada [21].

### Funding and ethics

Our study adhered to the Declaration of Helsinki – Ethical Principles for Research for Medical Research involving Human Participants. Research ethics board approval was obtained from the University of Ottawa (H-11-23-9815) and Public Health Agency of Canada (2023–034P). All participants provided signed informed consent. Funding was obtained from the National Microbiology Laboratory Branch. The clinical study ID for this research was NCT06586905.

## Results

### Participants

From August 26, 2024 to May 20, 2025, we performed 622 syphilis POCTs with matching serology on 600 participants. Most participants were male, MSM, and white (See Table 1).

**Table 1:** Participant characteristics

Metric		Number (#)	Percentage (%)
Sex	Male	514	82.6%
	Female	98	15.8%
	Trans	10	1.6%
Risk factors	MSM or trans	418	67.2%
	Injection drug use	11	1.8%
	Sex work	18	2.9%
Race/ethnicity	Arab	44	7.1%
	Black	95	15.3%
	East Asian	27	4.3%
	Indigenous	11	1.8%
	Latinx	28	4.5%
	South Asian	32	5.1%
	White	273	43.9%
	Not reported	112	18.0%

Regarding reasons for testing, 68.0% (n = 423/622) had presented to clinic for routine STI screening, 22.2% (n = 138/622) had syphilis-like symptoms, 6.4% (n = 40/622) were sexual contacts of someone recently diagnosed with syphilis, and 3.4% (n = 21/622) had serologic evidence of syphilis obtained elsewhere or earlier at this clinic and were seeking treatment.

### Test performance

Overall, for serology, 24.9% (n = 155/622) of tests had a reactive CMIA, of which 4 (0.64%) were false positive (as determined by RPR and TP.PA testing).^1^ True TP antibody prevalence in our cohort was 24.4% (n = 151/618). The RPR (any dilution) was reactive for 12.9% (n = 80/622) of tests. For POCT, we had 2 invalid results (0.3%, n = 2/622), and TP reactivity for 22.7% (n = 141/620) of tests and nTP reactivity for 11.8% (n = 73/620) of tests.

To determine the accuracy of this POCT, we compared the TP component of the POCT to the serologic CMIA, the CMIA and TP.PA combined, and the nTP component of the POCT to the RPR. (See Tables 2 and 3.) The POCT had sensitivities of 86.8% for the TP and 82.5% for the nTP and specificities of 97.9% for the TP and 99.1% for the nTP. (See Tables 4 and 5.) The POCT TP had a false positive rate of 2.2% (n = 10/465). Of these 10 participants with a false positive POCT TP result, 7 had presented with syphilis-like symptoms or as syphilis contacts and were treated per guidelines. The other 3 participants were not treated based on interpretation of a POCT TP reactive result and negative nTP result in an asymptomatic patient who was not a syphilis contact.

**Table 2:** Comparison of POCT TP results and CMIA serology results

Test	CMIA + ve	CMIA -ve	Total
POCT TP reactive	131	10^a^	141
POCT TP non-reactive	20^b^	455	475
Total	151	465	616^c^

^a^ False positive POCT TP results

^b^ False negative POCT TP results

^c^ From 622 tests but results from 6 tests were removed from the calculation (4 were CMIA false positive and 2 were giving invalid results in the POCT)

**Table 3:** Comparison of POCT nTP results and RPR serology results

Test	RPR reactive	RPR non-reactive	Total
POCT nTP reactive	66	5^*^	71
POCT nTP non-reactive	14^+^	531	545
Total	80	536	616^#^

^*^ False positive POCT nTP results

^+^ False negative POCT nTP results

^#^ From 622 tests but results from 6 tests were removed from the calculation (4 were CMIA false positive and 2 were giving invalid results in the POCT)

**Table 4:** POCT TP Performance

Statistics for TP	Value	95% CI
Sensitivity	86.8%	80.3% – 91.7%
Specificity	97.9%	96.1% –99.0%
Positive likelihood ratio	40.3	21.8–74.7
Negative likelihood ratio	0.14	0.1–0.2
Positive predictive value	92.9%	87.6% – 96.0%
Negative predictive value	95.8%	93.8% –97.2%

**Table 5:** POCT nTP performance

Statistics for nTP	Value	95% CI
Sensitivity	82.5%	72.4% –90.1%
Specificity	99.1%	97.8% – 99.7%
Positive likelihood ratio	88.4	36.8–212.9
Negative likelihood ratio	0.18	0.1–0.3
Positive predictive value	93.0%	84.6% – 97.0%
Negative predictive value	97.4%	95.9% – 98.4%

We recalculated the TP performance for the POCT using only serologic CMIA reactive results with a reactive TP.PA result (thus excluding inconclusive results where the TP.PA results were indeterminant). Such inconclusive results occurred in 9 serologic samples (1.4% of 622 tests done). This changed the denominator to 607 (607 = 622 total tests minus 4 false positive serologic results, 2 invalid POCT results, and 9 inconclusive serologic results). Removal of these cases increased the POCT TP sensitivity from 86.8% to 90.1%. See Tables 6 and 7.

**Table 6:** Comparison of POCT TP results and true positive CMIA serology results

Test	CMIA reactive	CMIA non-reactive	Total
POCT TP reactive	128	10^*^	138
POCT TP non-reactive	14^+^	455	469
Total	142	465	607^#^

^*^ False positive POCT nTP results

^+^ False negative POCT nTP results

^#^ From 622 tests but results from 15 tests were removed from the calculation (2 invalid POCT results & 4 false positive serology CMIA & 9 indeterminant TP.PA results)

**Table 7:** POCT TP performance

Statistic	Value	95% CI
Sensitivity	90.1%	84.0% – 94.5%
Specificity	97.9%	96.1% – 99.0%
Positive likelihood ratio	41.9	22.7–77.6
Negative likelihood ratio	0.1	0.1–0.2
Positive predictive value	92.8%	87.4% – 96.0%
Negative predictive value	97.0%	95.2% –98.2%

We also analyzed test sensitivity based on serologic RPR titers. See Table 8.

**Table 8:** POCT performance by RPR dIlution

RPR titer	# of POCT + ve for TP & nTP	# serology + ve for CMIA & RPR	Sensitivity of POCT compared to serology	95% CI
1:1	12	17	70.6%	44.0% – 89.7%
1:2	14	19	73.7%	48.8% – 90.9%
1:4	8	10	80.0%	44.4% – 97.5%
≥1:8	32	34	94.1%	80.3% – 99.3%
≥1:16	23	23	100.0%	85.7%–100.0%
1:1 to 1:4	34	46	73.9%	58.9% – 85.7%
Any titer	66	80	82.5%	72.4% – 90.1%

To answer our question regarding clinical outcomes, we reviewed the new infections in our sample, the number of infections identified by POCT and serology, and the number of clinical cases that were appropriately treated or not treated when the patient presented for care. From all 622 syphilis tests performed, there were 37 new infections that required treatment (positivity rate of 5.9%), of which 5.4% (n = 2/37) were diagnosed based on positive direct fluorescence antibody (DFA) testing (with negative serology) and 94.6% (n = 35/37) were diagnosed based on serology only. The POCT identified 86.5% (n = 32/37) of all new infections, and 91.4% (n = 32/35) of new infections identified by serology. Of the 3 infections identified by serology but not POCT, 2 were late latent infections (with a non-reactive RPR) and 1 was a new primary infection (with a reactive CMIA, non-reactive RPR, and a reactive DFA from a lesion). Of new diagnoses that were identified in our clinic, the POCT thus identified 97.0% (n = 32/33^2^) of infectious syphilis cases detected by serology and 100% (n = 32/32) of infectious syphilis cases where the RPR was reactive.

Of these 37 infections, 56.8% (n = 21/37) were returning to clinic for treatment based on positive test results from the preceding week. For the remaining 16 new infections, of which 87.5% (n = 14/16) were treated at the point of care, the reasons for testing were as follows: 43.8% (n = 7/16) had syphilis-like symptoms, 25.0% (n = 4/16) were syphilis contacts, and 31.3% (n = 5/16) were doing asymptomatic screening with POCT results positive for both TP and nTP antibodies. The POCT thus enabled us to immediately treat nearly one-third of the new infections we identified in our STI clinic, without requiring up to 7 days for serology results to return and an additional 1–3 days for the patient to return-to-clinic for treatment. In the other two-thirds of cases, clinical guidelines already recommended treatment (because these patients had syphilis-like symptoms or were syphilis contacts), meaning the decision to treat likely would not have varied in the absence of a reactive syphilis POCT.

The POCT also informed our clinicians’ decisions to withhold antibiotics. Among the 583^3^ tests we conducted when a syphilis infection was not identified, 20.4% (n = 119/583) were done on participants who had presented to clinic with symptoms suggestive of secondary syphilis (e.g., diffuse rash, mucus patches, possible condylomata lata). Based on United States guidelines [4], MSM with such symptoms should receive empiric treatment before serologic results become available. From these 119 patient encounters, we had 63 MSM patients with suggestive symptoms of secondary syphilis, for whom our clinicians used a non-reactive result (for those with no prior history of syphilis) or faintly reactive POCT nTP result (for persons with a prior history of syphilis who would likely retain low levels of residual non-treponemal activity, but be non-infectious) to correctly withhold treatment in 74.6% (n = 47/63) of cases. This decision making was based on two points: (1) that an nTP result should be present in virtually 100% of cases of secondary syphilis, and (2) that an nTP titer of ≥ 1:8 is usually present in secondary syphilis [1]. In all 47 cases, serology confirmed that there was no active syphilis infection requiring treatment.

## Discussion

From August 2024 – May 2025, we evaluated MedMira’s Multiplo® Complete Syphilis (TP/nTP) antibody POCT [15] compared to conventional serology in an STI clinic in Ottawa, Canada. We completed 622 syphilis POCT and serology tests on 600 participants. Compared to serology when the CMIA and TP.PA tests were reactive, the POCT TP had a sensitivity of 90.1% and specificity of 97.9%. Compared to RPR, the POCT nTP had a sensitivity of 82.5% and specificity of 99.1%. When we stratified the POCT nTP results based on RPR titers, the POCT nTP had a sensitivity of 94.1% for RPR dilutions ≥ 1:8 and 73.9% for RPR dilutions of 1:1–1:4. For clinical performance, compared to serology, the POCT identified 91.4% of new syphilis infections and 97% of primary, secondary, or early latent syphilis, as defined by Public Health Agency of Canada case definitions [14]. The POCT also identified 100% of cases of infectious syphilis when the RPR was reactive (any dilution). The POCT also allowed us to (1) immediately treat one-third of the new syphilis diagnoses we had during the study, and (2) correctly withhold treatment for 47 (or threequarters of) MSM patients who were identified as not having a new syphilis infection, but who had presented to clinic with syphilis risk factors and symptoms suggestive of secondary syphilis. These results raise a few points about the pearls and pitfalls of syphilis POCTs in an STI clinic.

Regarding the benefits of this device, our results show that dual TP/nTP POCTs can improve patient outcomes. The high TP sensitivity we observed (90.1%) and the high positive likelihood ratio (42), made this device useful to rule in infections in an urban STI clinic. Indeed, one-third of new diagnoses of infectious syphilis in our clinic were identified by nurses during routine screening of asymptomatic patients who did not report being syphilis contacts. In these cases, we had no clinical indication to treat for syphilis but did so immediately due to a positive POCT with reactive TP and nTP. This reduced turnaround time likely helped limit onward syphilis transmission due to rapid eradication of a transmissible infection [23], especially considering that people with infectious syphilis are rendered non-infectious ~ 24 hours after treatment [6]. However, if we had waited for serology results, treatment would have been delayed by over a week due to the time it takes to obtain results, and then contact, book in, and have a patient return to clinic. In the current context of increasing syphilis transmission in all populations, rapid diagnosis and treatment can help decrease transmission [24]. Rapid diagnosis and immediate treatment could also play a critical role in clinical management for patients when follow-up cannot be guaranteed [24, 25]. These finding also align with recent reviews [16, 23, 26–28], one small study on a different TP/nTP device in the Canadian Artic [29], and prior evaluations of the ChemBio DDP POCT [12–14].

Another benefit of the MedMira Multiplo® Complete Syphilis (TP/nTP) POCT [15] was the nTP component. With a specificity of ~ 99%, availability of a rapid nTP result allowed clinicians to withhold treatment when it was not required, although indicated based on clinical guidelines [6, 7] (e.g., MSM with a rash). This benefit cannot be understated. While much focus to date for syphilis POCTs has been on their ability to facilitate rapid treatment [21, 23, 26, 27, 30], our results highlight that these devices can promote antibiotic stewardship – and thus align with the work of Causer et al [12]. who identified that these devices could “[avoid] unnecessary treatment” by using them to withhold unneeded treatment when patients do not have an active syphilis infection. In addition to focusing on rapid eradication of syphilis through POCTs, the device we used thus helped avoid subjecting patients to inappropriate treatment in over threequarters of cases when current guidelines recommend antibiotic administration for MSM with symptoms of secondary syphilis.

Although reflexive empiric treatment for an infection like syphilis may be required to rein in its ongoing transmission, this will result in overtreatment, which will not be without the development of bystander resistance. In other words, due to the high transmissibility of syphilis and clinical complexities for diagnosis and staging, a low threshold to empirically treat is likely needed; however, this will approach will contribute to needless antibiotic usage. We feel that the control and management of infectious diseases should not be compartmentalized, such that we overtreat for syphilis at the expense of overall antimicrobial resistance. We therefore feel the ability to withhold treatment is a key consideration for the future evaluation and use of syphilis POCTs. Such an approach also aligns with recent updates to the British guideline for gonorrhea [31] and European guideline [32] for chlamydia, which now recommend more judicious use of antibiotics.

Our results did nevertheless highlight that syphilis POCTs do have pitfalls. These are not problems that render the devices non-useable; rather, they are limitations that – if/when addressed – would minimize harms and maximize benefits. For one, the utility of TP-only testing will diminish as TP antibody prevalence increases. As syphilis transmission continues, more people will have TP antibodies, which do not necessarily indicate an active transmissible infection, as these antibodies may be residual from historical treated infections [27]. In our cohort, ~ 25% of participants had TP antibodies, whereas only ~ 6% had a syphilis infection that needed treatment. This finding highlights that TP-only POCTs would be most useful in settings or populations with low prevalence, and less useful in settings where high rates of syphilis transmission have been ongoing for years (as is the case in our context). This finding also suggests that it may not be prudent to automatically administer syphilis treatment for persons with reactive POCT TP results. Further investigations – and the use of conventional serology to monitor post-treatment nTP antibody responses – are still required for any patient with a reactive result. It is important that nurses who intend on using syphilis POCTs in frontline urban STI clinics consider these points.

Moreover, it is important to remember that syphilis diagnosis is not based on laboratory results alone (whether conventional serology or POCT), but rather, such diagnosis is the outcome of patient history, physical examination, and test results [1, 6, 7]. For one, all TP antibody test results yield false positive results [8]. In our study, serology yielded false positive results at a rate of 0.64% (n = 4/622), compared to 1.6% (n = 10/620) for the POCT. Serology, moreover, yielded inconclusive results in another 1.4% (n = 9/622) of tests, for a combined false positive plus inconclusive rate of 2.1% (n = 13/622). Interpreting these test results as budding syphilis seroconversions versus historical treated infections with waning antibody levels versus false positive results can only occur in the context of a detailed patient history and physical examination [1]. Syphilis POCTs cannot replace clinical decision-making. They can however be useful clinical tools, which are used in conjunction with relevant direct testing for syphilis and serology to support shared decision-making between patients and healthcare providers during clinical visits. We found that the MedMira Multiplo® Complete Syphilis (TP/nTP) POCT [15] can be used in combination with our previously published syphilis staging algorithm [22] to make decisions about care.

Further supporting the need for patient history and examination is that 5.6% of syphilis diagnoses in our study arose from direct testing when serology was negative. When we take the 37 new diagnoses of syphilis in our study and exclude the 21 patients who participated when they returned to clinic for treatment (thus leaving 16 new infections diagnosed when we first ran the POCT and serology), direct testing identified 12.5% (n = 2/16) of these cases – which aligns with previous research on direct testing for syphilis [33–35]. The POCT, furthermore, while it detected 97% of primary, secondary, and early latent syphilis infections, missed 2 late latent infections, compared to serology. (See Box 1 for case definitions.) While late latent syphilis cannot be transmitted sexually, vertical transmission occurs at a rate of 10% [6]. In light of current increases in congenital syphilis (including its sequelae of deformity and death) [2–5], it is important to not over-rely on negative POCTs to curb vertical transmission. Conventional serology is still required, in combination with patient history and examination. The concern with syphilis POCTs is that their results can be difficult to interpret. Without clear guidelines on POCT use (e.g., interpretation algorithms), these devices could worsen syphilis transmission.

### Limitations

Our results must be interpreted considering certain limitations. First, our study occurred in one clinic in a city where high numbers of syphilis diagnoses have been occurring for many years, of which nearly one-third have occurred specifically within our STI clinic [2]. Syphilis seroprevalence will differ in other settings. Additionally, this prolonged period of managing high numbers of syphilis cases has fostered clinical expertise. POCT results might have yielded overtreatment (1) in cases of false positive results and (2) in cases of true positive results that aligned with historical treated infections in patients with possible symptoms of secondary syphilis. Second, during the study, the only direct testing available was DFA, which is ~ 75% sensitive compared to polymerase chain reaction testing [1]. We may have missed diagnosing some cases of primary syphilis. Third, none of our participants was pregnant. Before syphilis POCTs are used to direct treatment during pregnancy, trials need to include pregnant persons. Fourth, the device required operators who were familiar and confident in interpreting qualitative tests. Our TP and nTP reactive results varied in intensities, and a subset had additional flecks on the membrane. There were also multiple steps involved in this test, which may limit its use in non-clinical settings. Future research should involve less experienced operators, rather than licensed healthcare professionals who work in STI clinics.

## Conclusions

The rates of syphilis have risen with subsequent shifts in transmission among all groups [2–5]. Because syphilis POCTs can facilitate prompt treatment, we completed the first clinical trial of the unlicensed MedMira Multiplo® Complete Syphilis (TP/nTP) POCT [12] in an STI clinic in Ottawa, Canada and found the device to be a good addition to our clinical toolkit, especially due to the nTP availability. This aligns with previous research involving other dual TP/nTP syphilis POCTs [12–14]. The POCT we used enabled faster results for patients who would not have received empiric treatment during their visit. We also found that the POCT enabled clinicians to correctly withhold antibiotics, despite guideline recommendations. This highlights the role of syphilis POCTs to, not only identify new infections for immediate treatment, but also to enhance antibiotic stewardship by correctly withholding treatment during initial clinical visits. These findings also suggests that POCTs should be evaluated on their abilities to both rule in and rule out syphilis infections. These devices can then function to increase treatment and maximize antibiotic stewardship. The main conclusion from our study was that POCTs will yield the best outcomes when understood as clinical tools, not a panacea for ongoing increases in syphilis transmission. With good information on POCTs, and good data from milieux where these devices will be used (e.g., STI clinics), we can use them constructively, while minimizing the risk of missed diagnosis and overtreatment. The POCT we evaluated is one device that can help – but only when used appropriately.

## Electronic supplementary material

Below is the link to the electronic supplementary material.

Supplementary Material 1

Supplementary Material 2

## Data Availability

Included online with publication.

## Footnotes

1 All POCTs were TP and nTP negative for these 4 false positive serologic CMIA tests.

2 33 infectious cases = 37 total cases, minus 2 that were serologic negative with positive DFA results, and minus 2 infections that were late latent.

3 583 tests = 622 minus 37 positive cases, minus 2 invalid POCT results.
