# Supplementary material for: Evaluating the MedMira Multiplo® Complete Syphilis (TP/nTP) antibody test in a sexually transmitted infection clinic in Ottawa, Canada: increased rapid diagnosis and improved antibiotic stewardship
Source: BMC Infect Dis. 2025 Dec 8;26:48. doi: 10.1186/s12879-025-12263-w (PMC12797785; doi:10.1186/s12879-025-12263-w)

ABBREVIATION LIST

- **RPR**: Rapid plasma regain (non-treponemal syphilis test)
- **4-Fold:** 2-tube change in RPR dilution (e.g., from 1:8 to 1:32)
- **2-Fold:** 1-tube change in RPR dilution (e.g., from 1:8 to 1:16)
- **LFT**: Liver function test
- **DFA**: Direct Fluorescence Antibody test
- **PrEP**: HIV pre-exposure prophylaxis

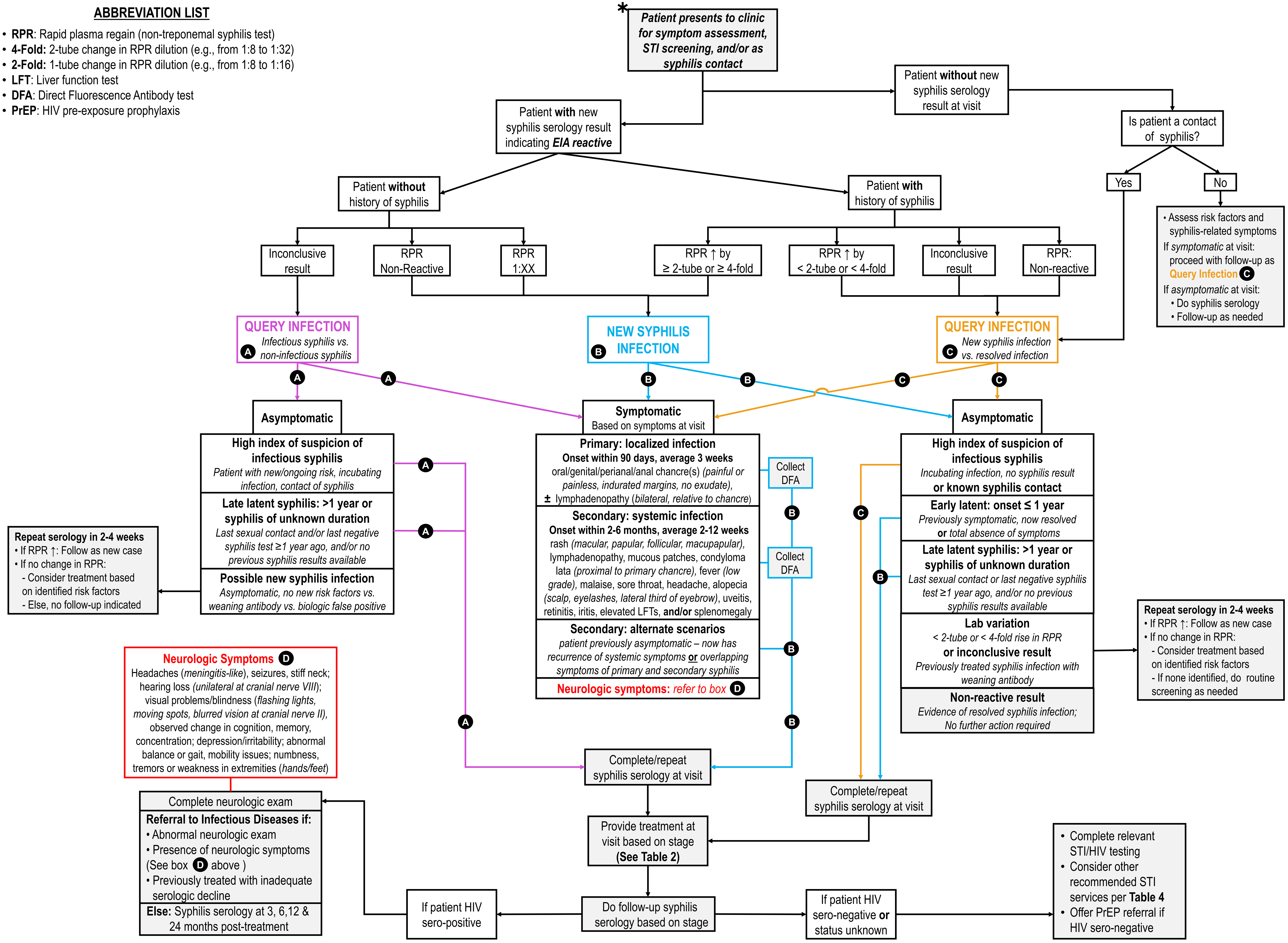

Supplement: Supplementary file 1 — Supplementary Material 1 [file 12879_2025_12263_MOESM1_ESM.pdf]
