# Supplementary material for: Evaluating the MedMira Multiplo® Complete Syphilis (TP/nTP) antibody test in a sexually transmitted infection clinic in Ottawa, Canada: increased rapid diagnosis and improved antibiotic stewardship
Source: BMC Infect Dis. 2025 Dec 8;26:48. doi: 10.1186/s12879-025-12263-w (PMC12797785; doi:10.1186/s12879-025-12263-w)
Supplement: Supplementary file 2 — Supplementary Material 2 [file 12879_2025_12263_MOESM2_ESM.pdf]

		POCT RESULTS			SYPHILIS SEROLOGY RESULTS			DIRECT TEST RESULTS	FINAL
**Sex**	**Reason for testing**	**TP Result**	**nTP result**	**Final Interpretation**	**CMIA**	**RPR**	**TPPA**	**Result**	**Final Case Interpretation**
Male	Syphilis symptoms	Non-Reactive	Non-Reactive	Negative	Non-Reactive				Negative (not a case)
Male	STI screening	Non-Reactive	Non-Reactive	Negative	Non-Reactive				Negative (not a case)
Male	STI screening	**Reactive**	Non-Reactive	**Positive**	**Reactive**	**1:8**			Previously treated
Male	STI screening	Non-Reactive	Non-Reactive	Negative	Non-Reactive				Negative (not a case)
Female	Syphilis symptoms	Non-Reactive	Non-Reactive	Negative	Non-Reactive				Negative (not a case)
Female	STI screening	Non-Reactive	Non-Reactive	Negative	Non-Reactive				Negative (not a case)
Male	STI screening	Non-Reactive	Non-Reactive	Negative	Non-Reactive				Negative (not a case)
Male	STI screening	Non-Reactive	Non-Reactive	Negative	Non-Reactive				Negative (not a case)
Female	STI screening	Non-Reactive	Non-Reactive	Negative	Non-Reactive				Negative (not a case)
Male	Syphilis symptoms	Non-Reactive	Non-Reactive	Negative	Non-Reactive				Negative (not a case)
Male	Syphilis symptoms	**Reactive**	**Reactive**	**Positive**	**Reactive**	**1:1**			Previously treated
Male	Syphilis symptoms	Non-Reactive	Non-Reactive	Negative	Non-Reactive				Negative (not a case)
Male	STI screening	Non-Reactive	Non-Reactive	Negative	Non-Reactive				Negative (not a case)
Female	Syphilis symptoms	Non-Reactive	Non-Reactive	Negative	Non-Reactive				Negative (not a case)
Male	STI screening	Non-Reactive	Non-Reactive	Negative	Non-Reactive				Negative (not a case)
Male	Syphilis symptoms	Non-Reactive	Non-Reactive	Negative	Non-Reactive				Negative (not a case)
Male	Syphilis symptoms	**Reactive**	Non-Reactive	**Positive**	**Reactive**	Non-Reactive	Previous Reactive		Previously treated
Male	Syphilis case	**Reactive**	**Reactive**	**Positive**	**Reactive**	**1:16**			**New infection**
Male	STI screening	Non-Reactive	Non-Reactive	Negative	**Reactive**	Non-Reactive	**Indeterminate**		Previously treated
Male	STI screening	Non-Reactive	Non-Reactive	Negative	Non-Reactive				Negative (not a case)
Male	STI screening	Non-Reactive	Non-Reactive	Negative	**Reactive**	Non-Reactive	**Indeterminate**		Previously treated
Male	STI screening	Non-Reactive	Non-Reactive	Negative	Non-Reactive				Negative (not a case)
Male	STI screening	Non-Reactive	Non-Reactive	Negative	Non-Reactive				Negative (not a case)
Male	STI screening	**Reactive**	Non-Reactive	**Positive**	**Reactive**	**1:1**			Previously treated, Lab variation
Male	Syphilis symptoms	**Reactive**	Non-Reactive	**Positive**	**Reactive**	**1:1**			Previously treated
Male	STI screening	Non-Reactive	Non-Reactive	Negative	Non-Reactive				Negative (not a case)
Male	STI screening	Non-Reactive	Non-Reactive	Negative	Non-Reactive				Negative (not a case)
Male	STI screening	Non-Reactive	Non-Reactive	Negative	Non-Reactive				Negative (not a case)
Male	STI screening	Non-Reactive	Non-Reactive	Negative	Non-Reactive				Negative (not a case)
Male	Syphilis symptoms	**Reactive**	Non-Reactive	**Positive**	Non-Reactive				**False positive**
Male	STI screening	Non-Reactive	Non-Reactive	Negative	Non-Reactive				Negative (not a case)
Male	STI screening	Non-Reactive	Non-Reactive	Negative	Non-Reactive				Negative (not a case)
Female	STI screening	Non-Reactive	Non-Reactive	Negative	Non-Reactive				Negative (not a case)
Male	Syphilis symptoms	Non-Reactive	Non-Reactive	Negative	Non-Reactive				Negative (not a case)
Male	Syphilis symptoms	Non-Reactive	Non-Reactive	Negative	Non-Reactive				Negative (not a case)

Female	STI screening	Non-Reactive	Non-Reactive	Negative	Non-Reactive				Negative (not a case)
Male	STI screening	Non-Reactive	Non-Reactive	Negative	Non-Reactive				Negative (not a case)
Male	Syphilis symptoms	**Reactive**	Non-Reactive	**Positive**	**Reactive**	Non-Reactive	**Reactive**		**New infection**
Female	Syphilis symptoms	Non-Reactive	Non-Reactive	Negative	Non-Reactive			**Positive**	**New infection**
Male	STI screening	Non-Reactive	Non-Reactive	Negative	Non-Reactive				Negative (not a case)
Female	STI screening	Non-Reactive	Non-Reactive	Negative	Non-Reactive				Negative (not a case)
Male	Syphilis symptoms	Non-Reactive	Non-Reactive	Negative	Non-Reactive				Negative (not a case)
Male	STI screening	Non-Reactive	Non-Reactive	Negative	Non-Reactive				Negative (not a case)
Male	STI screening	**Reactive**	**Reactive**	**Positive**	**Reactive**	**1:1**			Previously treated, Lab variation
Male	Syphilis symptoms	Non-Reactive	Non-Reactive	Negative	Non-Reactive				Negative (not a case)
Female	STI screening	Non-Reactive	Non-Reactive	Negative	Non-Reactive				Negative (not a case)
Female	STI screening	Non-Reactive	Non-Reactive	Negative	Non-Reactive				Negative (not a case)
Male	STI screening	Non-Reactive	Non-Reactive	Negative	Non-Reactive				Negative (not a case)
Female	STI screening	Non-Reactive	Non-Reactive	Negative	Non-Reactive				Negative (not a case)
Male	Syphilis symptoms	**Reactive**	**Reactive**	**Positive**	**Reactive**	**1:8**			Previously treated
Male	STI screening	Non-Reactive	Non-Reactive	Negative	Non-Reactive				Negative (not a case)
Male	STI screening	Non-Reactive	Non-Reactive	Negative	Non-Reactive				Negative (not a case)
Female	STI screening	Non-Reactive	Non-Reactive	Negative	Non-Reactive				Negative (not a case)
Male	Syphilis contact	**Reactive**	Non-Reactive	**Positive**	**Reactive**	Non-Reactive	Previous Reactive		Previously treated
Male	STI screening	Non-Reactive	Non-Reactive	Negative	**Reactive**	Non-Reactive	**Indeterminate**		Previously treated
Male	Syphilis symptoms	**Reactive**	**Reactive**	**Positive**	**Reactive**	**1:1**			Previously treated
Trans	STI screening	Non-Reactive	Non-Reactive	Negative	Non-Reactive				Negative (not a case)
Female	STI screening	Non-Reactive	Non-Reactive	Negative	Non-Reactive				Negative (not a case)
Male	STI screening	**Reactive**	**Reactive**	**Positive**	**Reactive**	**1:64**			**New infection**
Male	Syphilis symptoms	Non-Reactive	Non-Reactive	Negative	Non-Reactive			Negative	Negative (not a case)
Female	STI screening	Non-Reactive	Non-Reactive	Negative	Non-Reactive				Negative (not a case)
Female	STI screening	Non-Reactive	Non-Reactive	Negative	Non-Reactive				Negative (not a case)
Male	STI screening	Non-Reactive	Non-Reactive	Negative	Non-Reactive				Negative (not a case)
Male	STI screening	Non-Reactive	Non-Reactive	Negative	Non-Reactive				Negative (not a case)
Male	STI screening	**Reactive**	Non-Reactive	**Positive**	**Reactive**	Non-Reactive	**Reactive**		Previously treated
Male	Syphilis contact	Non-Reactive	Non-Reactive	Negative	Non-Reactive				Negative (not a case)
Male	STI screening	Non-Reactive	Non-Reactive	Negative	Non-Reactive				Negative (not a case)
Male	STI screening	Non-Reactive	Non-Reactive	Negative	Non-Reactive				Negative (not a case)
Female	STI screening	Non-Reactive	Non-Reactive	Negative	Non-Reactive				Negative (not a case)

Male	Syphilis symptoms	Non-Reactive	Non-Reactive	Negative	Non-Reactive				Negative (not a case)
Female	STI screening	Non-Reactive	Non-Reactive	Negative	Non-Reactive				Negative (not a case)
Male	Syphilis symptoms	Non-Reactive	Non-Reactive	Negative	Non-Reactive			Negative	Negative (not a case)
Male	Syphilis case	**Reactive**	**Reactive**	**Positive**	**Reactive**	**1:32**			**New infection**
Female	STI screening	Non-Reactive	Non-Reactive	Negative	Non-Reactive				Negative (not a case)
Male	STI screening	Non-Reactive	Non-Reactive	Negative	Non-Reactive				Negative (not a case)
Female	STI screening	Non-Reactive	Non-Reactive	Negative	Non-Reactive				Negative (not a case)
Male	STI screening	Non-Reactive	Non-Reactive	Negative	Non-Reactive				Negative (not a case)
Male	Syphilis symptoms	Non-Reactive	Non-Reactive	Negative	Non-Reactive			Negative	Negative (not a case)
Male	STI screening	**Reactive**	**Reactive**	**Positive**	**Reactive**	**1:1**			Previously treated
Male	Syphilis symptoms	Non-Reactive	Non-Reactive	Negative	Non-Reactive				Negative (not a case)
Male	STI screening	Non-Reactive	Non-Reactive	Negative	Non-Reactive				Negative (not a case)
Male	STI screening	Non-Reactive	Non-Reactive	Negative	Non-Reactive				Negative (not a case)
Male	STI screening	**Reactive**	**Reactive**	**Positive**	**Reactive**	**1:1**			Previously treated
Female	STI screening	Non-Reactive	Non-Reactive	Negative	Non-Reactive				Negative (not a case)
Male	STI screening	Non-Reactive	Non-Reactive	Negative	Non-Reactive				Negative (not a case)
Male	STI screening	**Reactive**	Non-Reactive	**Positive**	**Reactive**	Non-Reactive	Previous Reactive		Previously treated
Male	STI screening	**Reactive**	Non-Reactive	**Positive**	**Reactive**	Non-Reactive	Previous Reactive		Previously treated
Male	STI screening	Non-Reactive	Non-Reactive	Negative	Non-Reactive				Negative (not a case)
Male	STI screening	**Reactive**	**Reactive**	**Positive**	**Reactive**	**1:2**			**New infection**
Male	STI screening	Non-Reactive	Non-Reactive	Negative	Non-Reactive				Negative (not a case)
Male	Syphilis symptoms	**Reactive**	**Reactive**	**Positive**	**Reactive**	**1:2**		Negative x2	Previously treated
Male	STI screening	Non-Reactive	Non-Reactive	Negative	Non-Reactive				Negative (not a case)
Male	Syphilis case	**Reactive**	Non-Reactive	**Positive**	**Reactive**	Non-Reactive	Previous Reactive		**New infection**
Male	STI screening	Non-Reactive	Non-Reactive	Negative	Non-Reactive				Negative (not a case)
Male	STI screening	Non-Reactive	Non-Reactive	Negative	Non-Reactive				Negative (not a case)
Male	STI screening	**Reactive**	**Reactive**	**Positive**	**Reactive**	**1:8**			Previously treated
Male	STI screening	**Reactive**	**Reactive**	**Positive**	**Reactive**	Non-Reactive	Previous Reactive		Previously treated
Male	STI screening	Non-Reactive	Non-Reactive	Negative	Non-Reactive				Negative (not a case)
Male	Syphilis symptoms	Non-Reactive	Non-Reactive	Negative	Non-Reactive			Negative	Negative (not a case)
Male	STI screening	Non-Reactive	Non-Reactive	Negative	Non-Reactive				Negative (not a case)

Male	Syphilis case	**Reactive**	**Reactive**	**Positive**	**Reactive**	**1:8**			**New infection**
Male	STI screening	**Reactive**	Non-Reactive	**Positive**	**Reactive**	Non-Reactive	Previous Reactive		Previously treated
Male	STI screening	Non-Reactive	Non-Reactive	Negative	Non-Reactive				Negative (not a case)
Male	Syphilis symptoms	Non-Reactive	Non-Reactive	Negative	Non-Reactive				Negative (not a case)
Male	STI screening	**Reactive**	**Reactive**	**Positive**	**Reactive**	**1:1**			Previously treated
Male	STI screening	Non-Reactive	Non-Reactive	Negative	Non-Reactive				Negative (not a case)
Male	Syphilis symptoms	Non-Reactive	Non-Reactive	Negative	Non-Reactive				Negative (not a case)
Male	STI screening	Non-Reactive	Non-Reactive	Negative	Non-Reactive				Negative (not a case)
Male	Syphilis symptoms	**Reactive**	**Reactive**	**Positive**	**Reactive**	Non-Reactive	**Reactive**	Negative	Previously treated
Male	STI screening	Non-Reactive	Non-Reactive	Negative	Non-Reactive				Negative (not a case)
Male	STI screening	Non-Reactive	Non-Reactive	Negative	Non-Reactive				Negative (not a case)
Male	STI screening	Non-Reactive	Non-Reactive	Negative	**Reactive**	Non-Reactive	**Indeterminate**		**New infection**
Female	STI screening	Non-Reactive	Non-Reactive	Negative	Non-Reactive				Negative (not a case)
Male	STI screening	Non-Reactive	Non-Reactive	Negative	Non-Reactive				Negative (not a case)
Male	STI screening	Non-Reactive	Non-Reactive	Negative	Non-Reactive				Negative (not a case)
Male	STI screening	Non-Reactive	Non-Reactive	Negative	Non-Reactive				Negative (not a case)
Male	STI screening	Non-Reactive	Non-Reactive	Negative	Non-Reactive				Negative (not a case)
Male	Syphilis symptoms	Non-Reactive	Non-Reactive	Negative	Non-Reactive			Negative	Negative (not a case)
Trans	STI screening	Non-Reactive	Non-Reactive	Negative	Non-Reactive				Negative (not a case)
Male	STI screening	Non-Reactive	Non-Reactive	Negative	Non-Reactive				Negative (not a case)
Male	Syphilis symptoms	**Reactive**	Non-Reactive	Negative	**Reactive**	Non-Reactive	**Indeterminate**		Previously treated
Female	STI screening	Non-Reactive	Non-Reactive	Negative	Non-Reactive				Negative (not a case)
Male	STI screening	**Reactive**	**Reactive**	**Positive**	**Reactive**	**1:4**			Previously treated, Lab variation
Male	STI screening	Non-Reactive	Non-Reactive	Negative	Non-Reactive				Negative (not a case)
Male	Syphilis symptoms	**Reactive**	**Reactive**	**Positive**	**Reactive**	**1:4**			Previously treated
Male	STI screening	**Reactive**	Non-Reactive	**Positive**	Non-Reactive				**False positive**
Male	Syphilis symptoms	Non-Reactive	Non-Reactive	Negative	Non-Reactive				Negative (not a case)
Male	STI screening	Non-Reactive	Non-Reactive	Negative	**Reactive**	Non-Reactive	Previous Reactive		Previously treated
Male	STI screening	Non-Reactive	Non-Reactive	Negative	Non-Reactive				Negative (not a case)
Male	STI screening	Non-Reactive	Non-Reactive	Negative	Non-Reactive				Negative (not a case)
Male	STI screening	**Reactive**	Non-Reactive	**Positive**	**Reactive**	Non-Reactive	**Indeterminate**		Previously treated
Male	STI screening	Non-Reactive	Non-Reactive	Negative	Non-Reactive				Negative (not a case)

Male	Syphilis symptoms	Non-Reactive	Non-Reactive	Negative	**Reactive**	Non-Reactive	Previous Reactive		Previously treated
Male	STI screening	Non-Reactive	Non-Reactive	Negative	Non-Reactive				Negative (not a case)
Male	Syphilis contact	Non-Reactive	Non-Reactive	Negative	Non-Reactive				Negative (not a case)
Female	Syphilis symptoms	**Reactive**	**Reactive**	**Positive**	**Reactive**	**1:128**		**Positive**	**New infection**
Male	Syphilis symptoms	**Reactive**	**Reactive**	**Positive**	**Reactive**	**1:2**			Previously treated
Female	STI screening	Non-Reactive	Non-Reactive	Negative	Non-Reactive				Negative (not a case)
Male	Syphilis symptoms	**Reactive**	Non-Reactive	**Positive**	**Reactive**	Non-Reactive	**Reactive**		Previously treated
Male	Syphilis symptoms	Non-Reactive	Non-Reactive	Negative	Non-Reactive				Negative (not a case)
Female	Syphilis symptoms	**Reactive**	Non-Reactive	**Positive**	Non-Reactive				**False positive**
Male	Syphilis symptoms	Non-Reactive	Non-Reactive	Negative	Non-Reactive				Negative (not a case)
Male	STI screening	Non-Reactive	Non-Reactive	Negative	Non-Reactive				Negative (not a case)
Male	Syphilis symptoms	Non-Reactive	Non-Reactive	Negative	Non-Reactive				Negative (not a case)
Male	STI screening	Non-Reactive	Non-Reactive	Negative	Non-Reactive				Negative (not a case)
Male	STI screening	**Reactive**	Non-Reactive	**Positive**	**Reactive**	**1:2**			Previously treated
Male	STI screening	Non-Reactive	Non-Reactive	Negative	**Reactive**	Non-Reactive	**Indeterminate**		Previously treated
Male	STI screening	Non-Reactive	Non-Reactive	Negative	Non-Reactive				Negative (not a case)
Female	STI screening	Non-Reactive	Non-Reactive	Negative	Non-Reactive				Negative (not a case)
Female	STI screening	Non-Reactive	Non-Reactive	Negative	**Reactive**	Non-Reactive	Non-Reactive		**False positive**
Male	Syphilis symptoms	Non-Reactive	Non-Reactive	Negative	Non-Reactive				Negative (not a case)
Male	STI screening	Non-Reactive	Non-Reactive	Negative	Non-Reactive				Negative (not a case)
Male	Syphilis case	**Reactive**	Non-Reactive	**Positive**	**Reactive**	**1:4**			**New infection**
Female	STI screening	Non-Reactive	Non-Reactive	Negative	Non-Reactive				Negative (not a case)
Male	Syphilis contact	Non-Reactive	Non-Reactive	Negative	Non-Reactive				Negative (not a case)
Male	Syphilis case	**Reactive**	**Reactive**	**Positive**	**Reactive**	**1:8**			**New infection**
Male	Syphilis symptoms	Non-Reactive	Non-Reactive	Negative	Non-Reactive				Negative (not a case)
Male	STI screening	Non-Reactive	Non-Reactive	Negative	Non-Reactive				Negative (not a case)
Male	Syphilis symptoms	Non-Reactive	Non-Reactive	Negative	Non-Reactive				Negative (not a case)
Male	STI screening	**Reactive**	**Reactive**	**Positive**	**Reactive**	**1:2**			Previously treated
Male	Syphilis symptoms	Non-Reactive	Non-Reactive	Negative	Non-Reactive				Negative (not a case)

Male	Syphilis symptoms	**Reactive**	Non-Reactive	**Positive**	Non-Reactive			Negative	**False positive**
Male	STI screening	Non-Reactive	Non-Reactive	Negative	Non-Reactive				Negative (not a case)
Male	STI screening	**Reactive**	Non-Reactive	**Positive**	**Reactive**	Non-Reactive	Previous Reactive		Previously treated
Male	STI screening	Non-Reactive	Non-Reactive	Negative	Non-Reactive				Negative (not a case)
Male	STI screening	Non-Reactive	Non-Reactive	Negative	Non-Reactive				Negative (not a case)
Male	STI screening	**Reactive**	**Reactive**	**Positive**	**Reactive**	Non-Reactive	Previous Reactive		Previously treated
Female	Syphilis symptoms	Non-Reactive	Non-Reactive	Negative	Non-Reactive				Negative (not a case)
Male	Syphilis symptoms	**Reactive**	Non-Reactive	**Positive**	**Reactive**	Non-Reactive	**Indeterminate**		Previously treated
Male	Syphilis symptoms	Non-Reactive	Non-Reactive	Negative	Non-Reactive				Negative (not a case)
Male	STI screening	**Reactive**	Non-Reactive	**Positive**	**Reactive**	Non-Reactive	**Reactive**		Previously treated
Male	Syphilis symptoms	Non-Reactive	Non-Reactive	Negative	Non-Reactive				Negative (not a case)
Male	STI screening	Non-Reactive	Non-Reactive	Negative	Non-Reactive				Negative (not a case)
Female	STI screening	Non-Reactive	Non-Reactive	Negative	Non-Reactive				Negative (not a case)
Male	STI screening	Non-Reactive	Non-Reactive	Negative	Non-Reactive				Negative (not a case)
Male	STI screening	Non-Reactive	Non-Reactive	Negative	Non-Reactive				Negative (not a case)
Male	STI screening	Non-Reactive	Non-Reactive	Negative	Non-Reactive				Negative (not a case)
Male	STI screening	Non-Reactive	Non-Reactive	Negative	Non-Reactive				Negative (not a case)
Male	Syphilis contact	Non-Reactive	Non-Reactive	Negative	Non-Reactive				Negative (not a case)
Male	STI screening	Non-Reactive	Non-Reactive	Negative	Non-Reactive				Negative (not a case)
Male	STI screening	Non-Reactive	Non-Reactive	Negative	Non-Reactive				Negative (not a case)
Male	STI screening	Non-Reactive	Non-Reactive	Negative	Non-Reactive				Negative (not a case)
Male	STI screening	Non-Reactive	Non-Reactive	Negative	Non-Reactive				Negative (not a case)
Male	STI screening	Non-Reactive	Non-Reactive	Negative	Non-Reactive				Negative (not a case)
Male	STI screening	Non-Reactive	Non-Reactive	Negative	Non-Reactive				Negative (not a case)
Male	STI screening	**Reactive**	Non-Reactive	**Positive**	**Reactive**	Non-Reactive	Previous Reactive		Previously treated
Male	STI screening	Non-Reactive	Non-Reactive	Negative	Non-Reactive				Negative (not a case)
Male	STI screening	**Reactive**	**Reactive**	**Positive**	**Reactive**	Non-Reactive	Previous Reactive		Previously treated
Male	Syphilis symptoms	Non-Reactive	Non-Reactive	Negative	Non-Reactive			Negative	Negative (not a case)
Male	Syphilis symptoms	Non-Reactive	Non-Reactive	Negative	Non-Reactive			Negative	Negative (not a case)
Male	STI screening	Non-Reactive	Non-Reactive	Negative	Non-Reactive				Negative (not a case)
Female	Syphilis contact	Non-Reactive	Non-Reactive	Negative	Non-Reactive				Negative (not a case)
Male	STI screening	Non-Reactive	Non-Reactive	Negative	Non-Reactive				Negative (not a case)
Female	STI screening	Non-Reactive	Non-Reactive	Negative	Non-Reactive				Negative (not a case)
Male	STI screening	Non-Reactive	Non-Reactive	Negative	Non-Reactive				Negative (not a case)

Male	Syphilis contact	Non-Reactive	Non-Reactive	Negative	Non-Reactive				Negative (not a case)
Female	STI screening	Non-Reactive	Non-Reactive	Negative	Non-Reactive				Negative (not a case)
Male	STI screening	Non-Reactive	Non-Reactive	Negative	Non-Reactive				Negative (not a case)
Male	Syphilis symptoms	Non-Reactive	Non-Reactive	Negative	**Reactive**	Non-Reactive	**Reactive**	Positive	**New infection**
Male	STI screening	Non-Reactive	Non-Reactive	Negative	Non-Reactive				Negative (not a case)
Male	STI screening	Non-Reactive	Non-Reactive	Negative	Non-Reactive				Negative (not a case)
Male	STI screening	**Reactive**	Non-Reactive	**Positive**	**Reactive**	Non-Reactive	**Reactive**		Previously treated
Male	Syphilis contact	Non-Reactive	Non-Reactive	Negative	Non-Reactive				Negative (not a case)
Male	STI screening	**Reactive**	Non-Reactive	**Positive**	**Reactive**	Non-Reactive	Previous Reactive		Previously treated
Male	Syphilis symptoms	**Reactive**	**Reactive**	**Positive**	**Reactive**	**1:2**			Previously treated
Male	Syphilis symptoms	**Reactive**	**Reactive**	**Positive**	**Reactive**	**1:128**			**New infection**
Male	STI screening	**Reactive**	Non-Reactive	**Positive**	**Reactive**	**1:2**			Previously treated
Male	STI screening	Non-Reactive	Non-Reactive	Negative	Non-Reactive				Negative (not a case)
Male	Syphilis symptoms	Non-Reactive	Non-Reactive	Negative	Non-Reactive				Negative (not a case)
Male	Syphilis symptoms	Non-Reactive	Non-Reactive	Negative	Non-Reactive				Negative (not a case)
Male	STI screening	**Reactive**	**Reactive**	**Positive**	**Reactive**	**1:2**			Previously treated
Male	STI screening	Non-Reactive	Non-Reactive	Negative	Non-Reactive				Negative (not a case)
Male	Syphilis symptoms	**Reactive**	Non-Reactive	**Positive**	Non-Reactive			Negative	**False positive**
Male	Syphilis symptoms	Non-Reactive	Non-Reactive	Negative	Non-Reactive				Negative (not a case)
Female	STI screening	Non-Reactive	Non-Reactive	Negative	Non-Reactive				Negative (not a case)
Male	Syphilis case	**Reactive**	Non-Reactive	**Positive**	**Reactive**	**1:8**			**New infection**
Male	STI screening	Non-Reactive	Non-Reactive	Negative	Non-Reactive			Negative	Negative (not a case)
Male	Syphilis symptoms	**Reactive**	Non-Reactive	**Positive**	**Reactive**	Non-Reactive	Previous Reactive		Previously treated
Male	Syphilis symptoms	Non-Reactive	Non-Reactive	Negative	Non-Reactive				Negative (not a case)
Male	STI screening	Non-Reactive	Non-Reactive	Negative	Non-Reactive				Negative (not a case)
Male	STI screening	Non-Reactive	Non-Reactive	Negative	Non-Reactive				Negative (not a case)
Female	STI screening	Non-Reactive	Non-Reactive	Negative	**Reactive**	Non-Reactive	Previous Reactive		Previously treated
Female	STI screening	Non-Reactive	Non-Reactive	Negative	Non-Reactive				Negative (not a case)
Male	STI screening	Non-Reactive	Non-Reactive	Negative	Non-Reactive				Negative (not a case)
Male	STI screening	**Reactive**	**Reactive**	**Positive**	**Reactive**	**1:1**			Previously treated
Male	Syphilis symptoms	**Reactive**	Non-Reactive	**Positive**	**Reactive**	Non-Reactive	Previous Reactive		Previously treated

Trans	Syphilis symptoms	**Reactive**	**Reactive**	**Positive**	**Reactive**	**1:32**			**New infection**
Male	STI screening	Non-Reactive	Non-Reactive	Negative	Non-Reactive				Negative (not a case)
Male	STI screening	Non-Reactive	Non-Reactive	Negative	Non-Reactive				Negative (not a case)
Male	STI screening	Non-Reactive	Non-Reactive	Negative	Non-Reactive				Negative (not a case)
Male	Syphilis contact	Non-Reactive	Non-Reactive	Negative	Non-Reactive				Negative (not a case)
Male	STI screening	Non-Reactive	Non-Reactive	Negative	Non-Reactive				Negative (not a case)
Male	STI screening	Non-Reactive	Non-Reactive	Negative	Non-Reactive				Negative (not a case)
Male	STI screening	Non-Reactive	Non-Reactive	Negative	Non-Reactive				Negative (not a case)
Male	Syphilis symptoms	Non-Reactive	Non-Reactive	Negative	Non-Reactive				Negative (not a case)
Trans	STI screening	**Reactive**	**Reactive**	**Positive**	**Reactive**	**1:4**			Previously treated
Male	STI screening	Non-Reactive	Non-Reactive	Negative	Non-Reactive				Negative (not a case)
Male	STI screening	**Reactive**	**Reactive**	**Positive**	**Reactive**	**1:2**			Previously treated
Male	STI screening	**Reactive**	Non-Reactive	**Positive**	**Reactive**	Non-Reactive	Previous Reactive		Previously treated
Male	STI screening	Non-Reactive	Non-Reactive	Negative	Non-Reactive				Negative (not a case)
Male	Syphilis symptoms	Non-Reactive	Non-Reactive	Negative	Non-Reactive			Negative	Negative (not a case)
Male	STI screening	Non-Reactive	Non-Reactive	Negative	Non-Reactive				Negative (not a case)
Male	STI screening	Non-Reactive	Non-Reactive	Negative	Non-Reactive				Negative (not a case)
Male	STI screening	**Reactive**	Non-Reactive	**Positive**	**Reactive**	Non-Reactive	Previous Reactive		Previously treated
Male	STI screening	Non-Reactive	Non-Reactive	Negative	Non-Reactive				Negative (not a case)
Male	Syphilis contact	Non-Reactive	Non-Reactive	Negative	Non-Reactive				Negative (not a case)
Male	STI screening	Non-Reactive	Non-Reactive	Negative	Non-Reactive				Negative (not a case)
Male	STI screening	Non-Reactive	Non-Reactive	Negative	**Reactive**	Non-Reactive	Previous Reactive	Negative	Previously treated
Male	STI screening	**Reactive**	Non-Reactive	**Positive**	**Reactive**	Non-Reactive	Previous Reactive		Previously treated
Female	STI screening	Non-Reactive	Non-Reactive	Negative	Non-Reactive				Negative (not a case)
Male	STI screening	**Reactive**	**Reactive**	**Positive**	**Reactive**	Non-Reactive	**Reactive**		Previously treated
Male	Syphilis contact	Non-Reactive	Non-Reactive	Negative	Non-Reactive			**Positive**	**New infection**
Male	STI screening	Non-Reactive	Non-Reactive	Negative	Non-Reactive				Negative (not a case)
Male	Syphilis case	**Reactive**	**Reactive**	**Positive**	**Reactive**	**1:128**			**New infection**
Male	STI screening	Non-Reactive	Non-Reactive	Negative	Non-Reactive				Negative (not a case)
Male	Syphilis symptoms	Non-Reactive	Non-Reactive	Negative	Non-Reactive			Negative	Negative (not a case)
Male	Syphilis symptoms	Non-Reactive	Non-Reactive	Negative	Non-Reactive				Negative (not a case)
Male	STI screening	Non-Reactive	Non-Reactive	Negative	Non-Reactive				Negative (not a case)

Male	Syphilis symptoms	**Reactive**	**Reactive**	**Positive**	**Reactive**	**1:2**		Previously treated
Male	STI screening	**Reactive**	Non-Reactive	**Positive**	**Reactive**	Non-Reactive	**Reactive**	Previously treated
Male	STI screening	Non-Reactive	Non-Reactive	Negative	Non-Reactive			Negative (not a case)
Male	STI screening	Non-Reactive	Non-Reactive	Negative	Non-Reactive			Negative (not a case)
Male	Syphilis case	**Reactive**	**Reactive**	**Positive**	**Reactive**	**1:32**		**New infection**
Male	Syphilis contact	Non-Reactive	Non-Reactive	Negative	Non-Reactive			Negative (not a case)
Male	STI screening	Non-Reactive	Non-Reactive	Negative	Non-Reactive			Negative (not a case)
Male	STI screening	Non-Reactive	Non-Reactive	Negative	Non-Reactive			Negative (not a case)
Female	STI screening	Non-Reactive	Non-Reactive	Negative	Non-Reactive			Negative (not a case)
Female	STI screening	Non-Reactive	Non-Reactive	Negative	Non-Reactive			Negative (not a case)
Male	STI screening	Non-Reactive	Non-Reactive	Negative	Non-Reactive			Negative (not a case)
Male	Syphilis contact	Non-Reactive	Non-Reactive	Negative	Non-Reactive			Negative (not a case)
Male	STI screening	Non-Reactive	Non-Reactive	Negative	Non-Reactive			Negative (not a case)
Male	STI screening	Non-Reactive	Non-Reactive	Negative	Non-Reactive			Negative (not a case)
Male	STI screening	Non-Reactive	Non-Reactive	Negative	Non-Reactive			Negative (not a case)
Female	Syphilis contact	**Reactive**	Non-Reactive	**Positive**	Non-Reactive			**False positive**
Male	STI screening	Non-Reactive	Non-Reactive	Negative	Non-Reactive			Negative (not a case)
Male	Syphilis symptoms	**Reactive**	Non-Reactive	**Positive**	**Reactive**	**1:1**		Previously treated
Male	STI screening	Non-Reactive	Non-Reactive	Negative	Non-Reactive			Negative (not a case)
Male	STI screening	Non-Reactive	Non-Reactive	Negative	Non-Reactive			Negative (not a case)
Male	Syphilis case	**Reactive**	**Reactive**	**Positive**	**Reactive**	**1:32**		**New infection**
Male	STI screening	Non-Reactive	Non-Reactive	Negative	Non-Reactive			Negative (not a case)
Male	Syphilis contact	Non-Reactive	Non-Reactive	Negative	Non-Reactive			Negative (not a case)
Male	Syphilis symptoms	Non-Reactive	Non-Reactive	Negative	Non-Reactive			Negative (not a case)
Female	STI screening	Non-Reactive	Non-Reactive	Negative	Non-Reactive			Negative (not a case)
Male	STI screening	Non-Reactive	Non-Reactive	Negative	Non-Reactive			Negative (not a case)
Male	STI screening	Non-Reactive	Non-Reactive	Negative	Non-Reactive			Negative (not a case)
Male	STI screening	Non-Reactive	Non-Reactive	Negative	Non-Reactive			Negative (not a case)
Male	STI screening	Non-Reactive	Non-Reactive	Negative	Non-Reactive			Negative (not a case)
Male	STI screening	Non-Reactive	Non-Reactive	Negative	Non-Reactive			Negative (not a case)
Trans	STI screening	Non-Reactive	Non-Reactive	Negative	Non-Reactive			Negative (not a case)
Male	Syphilis symptoms	Non-Reactive	Non-Reactive	Negative	Non-Reactive			Negative (not a case)
Male	STI screening	Non-Reactive	Non-Reactive	Negative	Non-Reactive			Negative (not a case)
Male	STI screening	Non-Reactive	Non-Reactive	Negative	Non-Reactive			Negative (not a case)
Male	Syphilis contact	Non-Reactive	Non-Reactive	Negative	**Reactive**	Non-Reactive	Previous Reactive	Previously treated

Male	STI screening	Non-Reactive	Non-Reactive	Negative	Non-Reactive				Negative (not a case)
Male	Syphilis symptoms	Non-Reactive	Non-Reactive	Negative	Non-Reactive				Negative (not a case)
Male	STI screening	Non-Reactive	Non-Reactive	Negative	Non-Reactive				Negative (not a case)
Male	STI screening	Non-Reactive	Non-Reactive	Negative	Non-Reactive				Negative (not a case)
Male	STI screening	**Reactive**	**Reactive**	**Positive**	**Reactive**	**1:4**			Previously treated
Male	STI screening	Non-Reactive	Non-Reactive	Negative	Non-Reactive				Negative (not a case)
Trans	STI screening	Non-Reactive	Non-Reactive	Negative	Non-Reactive				Negative (not a case)
Male	Syphilis symptoms	Non-Reactive	Non-Reactive	Negative	Non-Reactive				Negative (not a case)
Male	STI screening	Non-Reactive	Non-Reactive	Negative	Non-Reactive				Negative (not a case)
Male	STI screening	Non-Reactive	Non-Reactive	Negative	Non-Reactive				Negative (not a case)
Male	STI screening	Non-Reactive	Non-Reactive	Negative	Non-Reactive				Negative (not a case)
Male	STI screening	**Reactive**	Non-Reactive	**Positive**	**Reactive**	Non-Reactive	Previous Reactive		Previously treated
Male	STI screening	Non-Reactive	Non-Reactive	Negative	Non-Reactive				Negative (not a case)
Male	Syphilis contact	**Reactive**	**Reactive**	**Positive**	**Reactive**	**1:64**			**New infection**
Female	Syphilis symptoms	Non-Reactive	Non-Reactive	Negative	Non-Reactive			Negative	Negative (not a case)
Male	Syphilis symptoms	Non-Reactive	Non-Reactive	Negative	Non-Reactive				Negative (not a case)
Male	STI screening	Non-Reactive	Non-Reactive	Negative	Non-Reactive				Negative (not a case)
Male	STI screening	Non-Reactive	Non-Reactive	Negative	Non-Reactive				Negative (not a case)
Male	STI screening	Non-Reactive	Non-Reactive	Negative	Non-Reactive				Negative (not a case)
Male	Syphilis contact	**Reactive**	Non-Reactive	**Positive**	**Reactive**	Non-Reactive	Previous Reactive		Previously treated
Male	STI screening	Non-Reactive	Non-Reactive	Negative	Non-Reactive				Negative (not a case)
Male	STI screening	Non-Reactive	Non-Reactive	Negative	Non-Reactive				Negative (not a case)
Male	STI screening	Non-Reactive	Non-Reactive	Negative	Non-Reactive				Negative (not a case)
Female	STI screening	Non-Reactive	Non-Reactive	Negative	Non-Reactive				Negative (not a case)
Male	STI screening	Non-Reactive	Non-Reactive	Negative	**Reactive**	Non-Reactive	**Reactive**		Previously treated
Male	STI screening	Non-Reactive	Non-Reactive	Negative	Non-Reactive				Negative (not a case)
Female	STI screening	Non-Reactive	Non-Reactive	Negative	Non-Reactive				Negative (not a case)
Male	STI screening	**Reactive**	Non-Reactive	**Positive**	**Reactive**	Non-Reactive	Previous Reactive		Previously treated
Male	STI screening	Non-Reactive	Non-Reactive	Negative	Non-Reactive				Negative (not a case)
Male	STI screening	**Reactive**	Non-Reactive	**Positive**	**Reactive**	Non-Reactive	Previous Reactive		Previously treated
Female	STI screening	Non-Reactive	Non-Reactive	Negative	Non-Reactive				Negative (not a case)
Female	STI screening	Non-Reactive	Non-Reactive	Negative	Non-Reactive				Negative (not a case)
Male	STI screening	Non-Reactive	Non-Reactive	Negative	Non-Reactive				Negative (not a case)
Trans	Syphilis symptoms	Non-Reactive	Non-Reactive	Negative	Non-Reactive				Negative (not a case)

Male	STI screening	Non-Reactive	Non-Reactive	Negative	Non-Reactive				Negative (not a case)
Male	STI screening	Non-Reactive	Non-Reactive	Negative	Non-Reactive				Negative (not a case)
Female	Syphilis symptoms	Non-Reactive	Non-Reactive	Negative	Non-Reactive				Negative (not a case)
Male	Syphilis symptoms	Non-Reactive	Non-Reactive	Negative	Non-Reactive				Negative (not a case)
Male	STI screening	Non-Reactive	Non-Reactive	Negative	Non-Reactive				Negative (not a case)
Male	STI screening	Non-Reactive	Non-Reactive	Negative	Non-Reactive				Negative (not a case)
Female	STI screening	Non-Reactive	Non-Reactive	Negative	Non-Reactive				Negative (not a case)
Male	STI screening	Non-Reactive	Non-Reactive	Negative	Non-Reactive				Negative (not a case)
Male	Syphilis symptoms	**Reactive**	Non-Reactive	**Positive**	**Reactive**	Non-Reactive	**Reactive**		Previously treated
Male	Syphilis symptoms	Non-Reactive	Non-Reactive	Negative	Non-Reactive				Negative (not a case)
Male	STI screening	Non-Reactive	Non-Reactive	Negative	Non-Reactive				Negative (not a case)
Female	STI screening	**Reactive**	Non-Reactive	**Positive**	**Reactive**	**1:4**			Previously treated
Male	STI screening	Non-Reactive	Non-Reactive	Negative	Non-Reactive				Negative (not a case)
Male	STI screening	Non-Reactive	Non-Reactive	Negative	Non-Reactive				Negative (not a case)
Male	Syphilis symptoms	Non-Reactive	Non-Reactive	Negative	Non-Reactive			Negative	Negative (not a case)
Male	Syphilis symptoms	Non-Reactive	Non-Reactive	Negative	Non-Reactive				Negative (not a case)
Male	STI screening	Non-Reactive	Non-Reactive	Negative	Non-Reactive				Negative (not a case)
Male	Syphilis contact	**Reactive**	**Reactive**	**Positive**	**Reactive**	**1:1**			Previously treated
Female	Syphilis symptoms	Non-Reactive	Non-Reactive	Negative	Non-Reactive				Negative (not a case)
Male	Syphilis symptoms	**Reactive**	**Reactive**	**Positive**	**Reactive**	**1:32**			Previously treated
Male	Syphilis contact	Non-Reactive	Non-Reactive	Negative	Non-Reactive				Negative (not a case)
Male	STI screening	Non-Reactive	Non-Reactive	Negative	Non-Reactive				Negative (not a case)
Male	Syphilis contact	**Reactive**	Non-Reactive	**Positive**	**Reactive**	Non-Reactive	Previous Reactive		Previously treated
Male	STI screening	Non-Reactive	Non-Reactive	Negative	Non-Reactive				Negative (not a case)
Female	STI screening	Non-Reactive	Non-Reactive	Negative	Non-Reactive				Negative (not a case)
Male	STI screening	**Reactive**	**Reactive**	**Positive**	**Reactive**	**1:2**			Previously treated
Male	STI screening	Non-Reactive	**Reactive**	**e**	Non-Reactive				Negative (not a case)
Male	STI screening	Non-Reactive	Non-Reactive	Negative	Non-Reactive				Negative (not a case)
Male	STI screening	Non-Reactive	Non-Reactive	Negative	Non-Reactive				Negative (not a case)
Male	STI screening	Non-Reactive	Non-Reactive	Negative	Non-Reactive				Negative (not a case)
Male	Syphilis case	**Reactive**	**Reactive**	**Positive**	**Reactive**	**1:64**			**New infection**
Male	STI screening	Non-Reactive	Non-Reactive	Negative	Non-Reactive				Negative (not a case)
Male	Syphilis symptoms	**Reactive**	**Reactive**	**Positive**	**Reactive**	**1:2**			Previously treated, Lab variation
Male	STI screening	Non-Reactive	Non-Reactive	Negative	Non-Reactive				Negative (not a case)

Male	STI screening	Non-Reactive	Non-Reactive	Negative	Non-Reactive				Negative (not a case)
Male	Syphilis symptoms	**Reactive**	Non-Reactive	**Positive**	**Reactive**	Non-Reactive	Previous Reactive		Previously treated
Male	Syphilis symptoms	Non-Reactive	Non-Reactive	Negative	Non-Reactive				Negative (not a case)
Male	STI screening	Non-Reactive	Non-Reactive	Negative	Non-Reactive				Negative (not a case)
Male	STI screening	Non-Reactive	Non-Reactive	Negative	Non-Reactive				Negative (not a case)
Male	Syphilis symptoms	Non-Reactive	Non-Reactive	Negative	Non-Reactive				Negative (not a case)
Male	STI screening	**Reactive**	Non-Reactive	**Positive**	**Reactive**	**1:2**			Previously treated
Male	Syphilis contact	Non-Reactive	Non-Reactive	Negative	Non-Reactive				Negative (not a case)
Male	STI screening	**Reactive**	**Reactive**	**Positive**	**Reactive**	**1:16**			Previously treated
Male	STI screening	Non-Reactive	Non-Reactive	Negative	Non-Reactive				Negative (not a case)
Male	STI screening	Non-Reactive	Non-Reactive	Negative	Non-Reactive				Negative (not a case)
Male	STI screening	Non-Reactive	Non-Reactive	Negative	Non-Reactive				Negative (not a case)
Male	STI screening	Non-Reactive	Non-Reactive	Negative	Non-Reactive				Negative (not a case)
Male	STI screening	Non-Reactive	Non-Reactive	Negative	Non-Reactive				Negative (not a case)
Female	STI screening	Non-Reactive	Non-Reactive	Negative	Non-Reactive				Negative (not a case)
Female	STI screening	**Reactive**	Non-Reactive	**Positive**	Non-Reactive				**False positive**
Female	STI screening	Non-Reactive	Non-Reactive	Negative	Non-Reactive				Negative (not a case)
Male	Syphilis case	**Reactive**	**Reactive**	**Positive**	**Reactive**	**1:128**		**Positive**	**New infection**
Male	STI screening	Non-Reactive	Non-Reactive	Negative	Non-Reactive				Negative (not a case)
Female	STI screening	Non-Reactive	Non-Reactive	Negative	Non-Reactive				Negative (not a case)
Male	STI screening	Non-Reactive	Non-Reactive	Negative	Non-Reactive				Negative (not a case)
Male	Syphilis contact	Non-Reactive	Non-Reactive	Negative	Non-Reactive			Negative	Negative (not a case)
Female	STI screening	Non-Reactive	Non-Reactive	Negative	Non-Reactive				Negative (not a case)
Male	STI screening	Non-Reactive	Non-Reactive	Negative	Non-Reactive				Negative (not a case)
Male	STI screening	**Reactive**	**Reactive**	**Positive**	**Reactive**	**1:4**			**New infection**
Male	Syphilis case	**Reactive**	**Reactive**	**Positive**	**Reactive**	**1:32**			**New infection**
Female	Syphilis symptoms	Non-Reactive	Non-Reactive	Negative	Non-Reactive				Negative (not a case)
Male	STI screening	**Reactive**	**Reactive**	**Positive**	**Reactive**	**1:16**			Previously treated
Male	Syphilis contact	**Reactive**	Non-Reactive	**Positive**	**Reactive**	Non-Reactive	Previous Reactive		Previously treated
Male	Syphilis symptoms	**Reactive**	Non-Reactive	**Positive**	Non-Reactive				**False positive**

Male	STI screening	Non-Reactive	Non-Reactive	Negative	Non-Reactive				Negative (not a case)
Male	STI screening	Non-Reactive	Non-Reactive	Negative	Non-Reactive				Negative (not a case)
Male	STI screening	Non-Reactive	Non-Reactive	Negative	Non-Reactive				Negative (not a case)
Male	Syphilis symptoms	Non-Reactive	Non-Reactive	Negative	Non-Reactive				Negative (not a case)
Female	STI screening	Non-Reactive	Non-Reactive	Negative	Non-Reactive				Negative (not a case)
Male	STI screening	Non-Reactive	Non-Reactive	Negative	Non-Reactive				Negative (not a case)
Female	Syphilis symptoms	Non-Reactive	Non-Reactive	Negative	Non-Reactive				Negative (not a case)
Male	STI screening	Non-Reactive	Non-Reactive	Negative	Non-Reactive				Negative (not a case)
Male	Syphilis symptoms	Non-Reactive	Non-Reactive	Negative	Non-Reactive				Negative (not a case)
Male	STI screening	Non-Reactive	Non-Reactive	Negative	Non-Reactive				Negative (not a case)
Female	STI screening	Non-Reactive	Non-Reactive	Negative	Non-Reactive				Negative (not a case)
Male	STI screening	Non-Reactive	Non-Reactive	Negative	Non-Reactive				Negative (not a case)
Male	STI screening	Non-Reactive	Non-Reactive	Negative	Non-Reactive				Negative (not a case)
Female	STI screening	Non-Reactive	Non-Reactive	Negative	Non-Reactive				Negative (not a case)
Male	STI screening	**Reactive**	Non-Reactive	**Positive**	**Reactive**	Non-Reactive	**Reactive**		Previously treated
Male	STI screening	Non-Reactive	Non-Reactive	Negative	Non-Reactive				Negative (not a case)
Male	STI screening	Non-Reactive	Non-Reactive	Negative	Non-Reactive				Negative (not a case)
Male	Syphilis symptoms	Non-Reactive	Non-Reactive	Negative	Non-Reactive				Negative (not a case)
Male	Syphilis case	**Reactive**	**Reactive**	**Positive**	**Reactive**	**1:8**			**New infection**
Male	Syphilis symptoms	Non-Reactive	Non-Reactive	Negative	**Reactive**	Non-Reactive	**Indeterminate**		Previously treated
Male	Syphilis symptoms	Non-Reactive	Non-Reactive	Negative	Non-Reactive				Negative (not a case)
Male	STI screening	Non-Reactive	Non-Reactive	Negative	Non-Reactive				Negative (not a case)
Female	STI screening	Non-Reactive	Non-Reactive	Negative	Non-Reactive				Negative (not a case)
Male	Syphilis case	**Reactive**	**Reactive**	**Positive**	**Reactive**	**1:16**			**New infection**
Male	Syphilis symptoms	**Reactive**	Non-Reactive	**Positive**	**Reactive**	Non-Reactive	**Reactive**	Negative	Previously treated
Male	STI screening	Non-Reactive	Non-Reactive	Negative	Non-Reactive				Negative (not a case)
Male	STI screening	Non-Reactive	Non-Reactive	Negative	Non-Reactive				Negative (not a case)
Male	STI screening	Non-Reactive	Non-Reactive	Negative	Non-Reactive				Negative (not a case)
Male	Syphilis symptoms	Non-Reactive	Non-Reactive	Negative	Non-Reactive				Negative (not a case)
Female	Syphilis contact	Non-Reactive	Non-Reactive	Negative	Non-Reactive				Negative (not a case)
Male	STI screening	Non-Reactive	Non-Reactive	Negative	Non-Reactive				Negative (not a case)
Male	STI screening	Non-Reactive	Non-Reactive	Negative	Non-Reactive				Negative (not a case)
Male	STI screening	Non-Reactive	Non-Reactive	Negative	Non-Reactive				Negative (not a case)
Male	Syphilis symptoms	**Reactive**	Non-Reactive	**Positive**	**Reactive**	Non-Reactive	Previous Reactive		Previously treated
Trans	STI screening	**Reactive**	Non-Reactive	**Positive**	**Reactive**	**1:2**			Previously treated

Male	STI screening	Non-Reactive	Non-Reactive	Negative	Non-Reactive			Negative (not a case)
Trans	STI screening	Non-Reactive	Non-Reactive	Negative	Non-Reactive			Negative (not a case)
Male	STI screening	Non-Reactive	Non-Reactive	Negative	Non-Reactive			Negative (not a case)
Male	STI screening	Non-Reactive	Non-Reactive	Negative	Non-Reactive			Negative (not a case)
Male	STI screening	Non-Reactive	Non-Reactive	Negative	**Reactive**	Non-Reactive	Previous Reactive	Previously treated
Male	STI screening	Non-Reactive	Non-Reactive	Negative	**Reactive**	Non-Reactive	Previous Reactive	Previously treated
Male	Syphilis symptoms	**Reactive**	Non-Reactive	**Positive**	**Reactive**	Non-Reactive	Previous Reactive	Previously treated
Male	Syphilis contact	**Reactive**	**Reactive**	**Positive**	**Reactive**	**1:16**		**New infection**
Male	Syphilis case	**Reactive**	**Reactive**	**Positive**	**Reactive**	**1:16**		**New infection**
Trans	Syphilis symptoms	Non-Reactive	Non-Reactive	Negative	Non-Reactive			Negative (not a case)
Male	STI screening	Non-Reactive	Non-Reactive	Negative	Non-Reactive			Negative (not a case)
Male	STI screening	**Reactive**	Non-Reactive	**Positive**	**Reactive**	Non-Reactive	**Reactive**	Previously treated
Male	Syphilis contact	**Reactive**	Non-Reactive	**Positive**	**Reactive**	Non-Reactive	Previous Reactive	Previously treated
Male	STI screening	**Reactive**	Non-Reactive	**Positive**	**Reactive**	Non-Reactive	**Reactive**	Previously treated
Male	STI screening	**Reactive**	**Reactive**	**Positive**	**Reactive**	**1:8**	Previous Reactive	**New infection**
Female	Syphilis case	Non-Reactive	Non-Reactive	Negative	**Reactive**	Non-Reactive	Previous Reactive	**New infection**
Male	STI screening	Non-Reactive	Non-Reactive	Negative	Non-Reactive			Negative (not a case)
Male	STI screening	Non-Reactive	Non-Reactive	Negative	Non-Reactive			Negative (not a case)
Male	Syphilis symptoms	**Reactive**	Non-Reactive	**Positive**	**Reactive**	Non-Reactive	**Reactive**	Previously treated
Male	Syphilis symptoms	Non-Reactive	Non-Reactive	Negative	Non-Reactive			Negative (not a case)
Male	Syphilis contact	Non-Reactive	Non-Reactive	Negative	Non-Reactive			Negative (not a case)
Male	Syphilis symptoms	Non-Reactive	Non-Reactive	Negative	Non-Reactive			Negative (not a case)
Male	Syphilis contact	**Reactive**	Non-Reactive	**Positive**	Non-Reactive			**False positive**
Male	STI screening	**Reactive**	**Reactive**	**Positive**	**Reactive**	**1:2**	Previous Reactive	Previously treated
Male	STI screening	Non-Reactive	Non-Reactive	Negative	Non-Reactive			Negative (not a case)
Male	STI screening	Non-Reactive	Non-Reactive	Negative	Non-Reactive			Negative (not a case)
Male	STI screening	Non-Reactive	Non-Reactive	Negative	Non-Reactive			Negative (not a case)
Male	Syphilis contact	Non-Reactive	Non-Reactive	Negative	Non-Reactive			Negative (not a case)
Female	Syphilis symptoms	Non-Reactive	Non-Reactive	Negative	Non-Reactive			Negative (not a case)

Male	STI screening	Non-Reactive	Non-Reactive	Negative	Non-Reactive				Negative (not a case)
Male	STI screening	Non-Reactive	Non-Reactive	Negative	Non-Reactive				Negative (not a case)
Female	Syphilis contact	Non-Reactive	Non-Reactive	Negative	Non-Reactive				Negative (not a case)
Male	STI screening	Non-Reactive	Non-Reactive	Negative	Non-Reactive				Negative (not a case)
Male	Syphilis symptoms	Non-Reactive	Non-Reactive	Negative	Non-Reactive				Negative (not a case)
Male	STI screening	Non-Reactive	Non-Reactive	Negative	Non-Reactive				Negative (not a case)
Male	Syphilis contact	Non-Reactive	Non-Reactive	Negative	Non-Reactive				Negative (not a case)
Male	Syphilis symptoms	Non-Reactive	Non-Reactive	Negative	Non-Reactive				Negative (not a case)
Female	STI screening	Non-Reactive	Non-Reactive	Negative	Non-Reactive				Negative (not a case)
Male	STI screening	**Reactive**	**Reactive**	**Positive**	**Reactive**	**1:1**	Previous Reactive		Previously treated
Male	STI screening	**Reactive**	Non-Reactive	**Positive**	**Reactive**	**1:2**			Previously treated
Male	STI screening	Non-Reactive	Non-Reactive	Negative	Non-Reactive				Negative (not a case)
Male	STI screening	**Reactive**	Non-Reactive	**Positive**	**Reactive**	**1:1**			Previously treated
Male	Syphilis symptoms	Non-Reactive	Non-Reactive	Negative	Non-Reactive				Negative (not a case)
Male	STI screening	Non-Reactive	Non-Reactive	Negative	Non-Reactive				Negative (not a case)
Male	STI screening	Non-Reactive	Non-Reactive	Negative	Non-Reactive				Negative (not a case)
Male	Syphilis symptoms	**Reactive**	**Reactive**	**Positive**	**Reactive**	**1:1**	Previous Reactive		Previously treated
Male	STI screening	Non-Reactive	Non-Reactive	Negative	Non-Reactive				Negative (not a case)
Male	STI screening	**Reactive**	**Reactive**	**Positive**	**Reactive**	**1:4**			Previously treated
Male	STI screening	Non-Reactive	Non-Reactive	Negative	Non-Reactive				Negative (not a case)
Male	Syphilis symptoms	Non-Reactive	Non-Reactive	Negative	**Reactive**	Non-Reactive	Previous Reactive		Previously treated
Female	STI screening	Non-Reactive	Non-Reactive	Negative	Non-Reactive				Negative (not a case)
Male	STI screening	Non-Reactive	Non-Reactive	Negative	Non-Reactive				Negative (not a case)
Male	Syphilis symptoms	Non-Reactive	Non-Reactive	Negative	Non-Reactive				Negative (not a case)
Male	STI screening	Non-Reactive	Non-Reactive	Negative	Non-Reactive				Negative (not a case)
Male	STI screening	Non-Reactive	Non-Reactive	Negative	Non-Reactive				Negative (not a case)
Male	STI screening	Non-Reactive	Non-Reactive	Negative	Non-Reactive				Negative (not a case)
Male	STI screening	**Reactive**	Non-Reactive	**Positive**	**Reactive**	**1:1**			Previously treated
Male	STI screening	Non-Reactive	Non-Reactive	Negative	Non-Reactive				Negative (not a case)
Male	STI screening	Non-Reactive	Non-Reactive	Negative	Non-Reactive				Negative (not a case)
Female	STI screening	Non-Reactive	Non-Reactive	Negative	Non-Reactive				Negative (not a case)
Male	Syphilis case	**Reactive**	Non-Reactive	**Positive**	**Reactive**	Non-Reactive	Previous Reactive	Negative	**New infection**
Male	STI screening	Non-Reactive	Non-Reactive	Negative	Non-Reactive				Negative (not a case)
Male	STI screening	Non-Reactive	Non-Reactive	Negative	Non-Reactive				Negative (not a case)

Male	STI screening	Non-Reactive	Non-Reactive	Negative	Non-Reactive				Negative (not a case)
Male	STI screening	Non-Reactive	Non-Reactive	Negative	Non-Reactive				Negative (not a case)
Female	STI screening	Non-Reactive	Non-Reactive	Negative	Non-Reactive				Negative (not a case)
Female	STI screening	Non-Reactive	Non-Reactive	Negative	Non-Reactive				Negative (not a case)
Female	STI screening	Non-Reactive	Non-Reactive	Negative	Non-Reactive				Negative (not a case)
Male	STI screening	Non-Reactive	Non-Reactive	Negative	Non-Reactive				Negative (not a case)
Male	Syphilis symptoms	Non-Reactive	Non-Reactive	Negative	Non-Reactive				Negative (not a case)
Female	STI screening	Non-Reactive	Non-Reactive	Negative	Non-Reactive				Negative (not a case)
Male	STI screening	**Reactive**	Non-Reactive	**Positive**	**Reactive**	Non-Reactive	Previous Reactive		Previously treated
Male	Syphilis symptoms	Non-Reactive	Non-Reactive	Negative	Non-Reactive			Negative	Negative (not a case)
Male	STI screening	Non-Reactive	Non-Reactive	Negative	Non-Reactive				Negative (not a case)
Female	Syphilis symptoms	Non-Reactive	Non-Reactive	Negative	Non-Reactive				Negative (not a case)
Male	Syphilis symptoms	Non-Reactive	Non-Reactive	Negative	**Reactive**	Non-Reactive	Non-Reactive		**False positive**
Male	STI screening	Non-Reactive	Non-Reactive	Negative	Non-Reactive				Negative (not a case)
Male	STI screening	Non-Reactive	Non-Reactive	Negative	Non-Reactive				Negative (not a case)
Female	Syphilis symptoms	Non-Reactive	Non-Reactive	Negative	**Reactive**	Non-Reactive	Non-Reactive		**False positive**
Male	STI screening	Non-Reactive	Non-Reactive	Negative	Non-Reactive				Negative (not a case)
Male	Syphilis contact	Non-Reactive	Non-Reactive	Negative	**Reactive**	Non-Reactive	Previous Reactive		Previously treated
Male	STI screening	Non-Reactive	Non-Reactive	Negative	Non-Reactive				Negative (not a case)
Male	STI screening	Non-Reactive	Non-Reactive	Negative	Non-Reactive				Negative (not a case)
Male	Syphilis symptoms	Non-Reactive	Non-Reactive	Negative	Non-Reactive			Negative	Negative (not a case)
Female	STI screening	Non-Reactive	Non-Reactive	Negative	Non-Reactive				Negative (not a case)
Male	Syphilis symptoms	**Reactive**	**Reactive**	**Positive**	**Reactive**	**1:1**	Previous Reactive		Previously treated
Male	Syphilis symptoms	Non-Reactive	Non-Reactive	Negative	Non-Reactive			Negative	Negative (not a case)
Male	Syphilis symptoms	**Reactive**	**Reactive**	**Positive**	**Reactive**	**1:2**			Previously treated
Male	STI screening	Non-Reactive	Non-Reactive	Negative	Non-Reactive				Negative (not a case)
Male	STI screening	**Reactive**	**Reactive**	**Positive**	**Reactive**	**1:8**	Previous Reactive	Negative	Previously treated
Male	STI screening	Non-Reactive	Non-Reactive	Negative	Non-Reactive				Negative (not a case)
Male	Syphilis symptoms	Non-Reactive	Non-Reactive	Negative	Non-Reactive				Negative (not a case)
Male	STI screening	Non-Reactive	Non-Reactive	Negative	Non-Reactive				Negative (not a case)
Female	STI screening	Non-Reactive	Non-Reactive	Negative	Non-Reactive				Negative (not a case)

Male	Syphilis symptoms	Non-Reactive	Non-Reactive	Negative	Non-Reactive		Negative	Negative (not a case)
Male	STI screening	Non-Reactive	Non-Reactive	Negative	Non-Reactive			Negative (not a case)
Male	STI screening	Non-Reactive	Non-Reactive	Negative	Non-Reactive			Negative (not a case)
Female	Syphilis symptoms	Non-Reactive	Non-Reactive	Negative	Non-Reactive			Negative (not a case)
Male	Syphilis contact	Non-Reactive	Non-Reactive	Negative	Non-Reactive			Negative (not a case)
Male	STI screening	Non-Reactive	Non-Reactive	Negative	Non-Reactive			Negative (not a case)
Male	Syphilis contact	Non-Reactive	Non-Reactive	Negative	Non-Reactive			Negative (not a case)
Male	STI screening	**Invalid**	**Invalid**	**Invalid/Indeterminate**	Non-Reactive			Negative (not a case)
Male	STI screening	Non-Reactive	Non-Reactive	Negative	Non-Reactive			Negative (not a case)
Male	STI screening	Non-Reactive	Non-Reactive	Negative	Non-Reactive			Negative (not a case)
Male	STI screening	Non-Reactive	Non-Reactive	Negative	Non-Reactive			Negative (not a case)
Male	STI screening	Non-Reactive	Non-Reactive	Negative	Non-Reactive			Negative (not a case)
Female	Syphilis case	**Reactive**	**Reactive**	**Positive**	**Reactive**	**1:64**		**New infection**
Male	STI screening	Non-Reactive	Non-Reactive	Negative	Non-Reactive			Negative (not a case)
Male	STI screening	Non-Reactive	Non-Reactive	Negative	Non-Reactive			Negative (not a case)
Male	STI screening	Non-Reactive	Non-Reactive	Negative	Non-Reactive			Negative (not a case)
Male	Syphilis case	**Reactive**	**Reactive**	**Positive**	**Reactive**	**1:16**		**New infection**
Male	STI screening	Non-Reactive	Non-Reactive	Negative	Non-Reactive			Negative (not a case)
Male	Syphilis symptoms	Non-Reactive	Non-Reactive	Negative	Non-Reactive			Negative (not a case)
Male	STI screening	Non-Reactive	Non-Reactive	Negative	Non-Reactive			Negative (not a case)
Male	Syphilis symptoms	Non-Reactive	Non-Reactive	Negative	Non-Reactive			Negative (not a case)
Male	STI screening	Non-Reactive	Non-Reactive	Negative	Non-Reactive			Negative (not a case)
Male	STI screening	Non-Reactive	Non-Reactive	Negative	Non-Reactive			Negative (not a case)
Male	Syphilis contact	**Reactive**	**Reactive**	**Positive**	**Reactive**	**1:1**		Previously treated
Male	Syphilis contact	Non-Reactive	Non-Reactive	Negative	Non-Reactive			Negative (not a case)
Male	Syphilis symptoms	Non-Reactive	Non-Reactive	Negative	Non-Reactive			Negative (not a case)
Male	STI screening	Non-Reactive	Non-Reactive	Negative	Non-Reactive			Negative (not a case)
Male	STI screening	Non-Reactive	Non-Reactive	Negative	Non-Reactive			Negative (not a case)
Male	STI screening	Non-Reactive	Non-Reactive	Negative	Non-Reactive			Negative (not a case)
Male	STI screening	Non-Reactive	Non-Reactive	Negative	Non-Reactive			Negative (not a case)
Male	STI screening	Non-Reactive	Non-Reactive	Negative	Non-Reactive			Negative (not a case)
Male	STI screening	Non-Reactive	Non-Reactive	Negative	Non-Reactive			Negative (not a case)
Female	STI screening	Non-Reactive	Non-Reactive	Negative	Non-Reactive			Negative (not a case)
Male	Syphilis symptoms	Non-Reactive	Non-Reactive	Negative	Non-Reactive			Negative (not a case)

Female	STI screening	Non-Reactive	Non-Reactive	Negative	Non-Reactive			Negative (not a case)
Female	STI screening	Non-Reactive	Non-Reactive	Negative	Non-Reactive			Negative (not a case)
Male	Syphilis symptoms	**Reactive**	**Reactive**	**Positive**	**Reactive**	**1:128**		**New infection**
Female	STI screening	Non-Reactive	Non-Reactive	Negative	Non-Reactive			Negative (not a case)
Male	STI screening	Non-Reactive	Non-Reactive	Negative	Non-Reactive			Negative (not a case)
Male	Syphilis symptoms	Non-Reactive	Non-Reactive	Negative	Non-Reactive			Negative (not a case)
Male	Syphilis contact	Non-Reactive	Non-Reactive	Negative	Non-Reactive			Negative (not a case)
Female	STI screening	Non-Reactive	Non-Reactive	Negative	Non-Reactive			Negative (not a case)
Male	STI screening	Non-Reactive	Non-Reactive	Negative	Non-Reactive			Negative (not a case)
Male	Syphilis case	**Reactive**	**Reactive**	**Positive**	**Reactive**	**1:32**		**New infection**
Female	STI screening	Non-Reactive	Non-Reactive	Negative	Non-Reactive			Negative (not a case)
Male	STI screening	Non-Reactive	Non-Reactive	Negative	**Reactive**	Non-Reactive	**Reactive**	Previously treated
Male	Syphilis symptoms	Non-Reactive	Non-Reactive	Negative	Non-Reactive			Negative (not a case)
Male	STI screening	**Reactive**	Non-Reactive	**Positive**	**Reactive**	Non-Reactive	**Reactive**	Previously treated
Male	STI screening	Non-Reactive	Non-Reactive	Negative	Non-Reactive			Negative (not a case)
Male	STI screening	Non-Reactive	Non-Reactive	Negative	Non-Reactive			Negative (not a case)
Male	STI screening	**Reactive**	**Reactive**	**Positive**	**Reactive**	**1:8**	Previous Reactive	Previously treated
Female	STI screening	Non-Reactive	Non-Reactive	Negative	Non-Reactive			Negative (not a case)
Male	STI screening	Non-Reactive	Non-Reactive	Negative	Non-Reactive			Negative (not a case)
Male	STI screening	Non-Reactive	Non-Reactive	Negative	Non-Reactive			Negative (not a case)
Male	Syphilis symptoms	Non-Reactive	Non-Reactive	Negative	**Reactive**	Non-Reactive	Previous Reactive	Previously treated
Female	STI screening	**Reactive**	Non-Reactive	**Positive**	Non-Reactive			**False positive**
Male	STI screening	Non-Reactive	Non-Reactive	Negative	Non-Reactive			Negative (not a case)
Male	STI screening	**Reactive**	**Reactive**	**Positive**	**Reactive**	**1:4**		Previously treated
Male	STI screening	Non-Reactive	Non-Reactive	Negative	Non-Reactive			Negative (not a case)
Male	STI screening	Non-Reactive	Non-Reactive	Negative	Non-Reactive			Negative (not a case)
Male	STI screening	Non-Reactive	Non-Reactive	Negative	Non-Reactive			Negative (not a case)
Male	STI screening	Non-Reactive	Non-Reactive	Negative	Non-Reactive			Negative (not a case)

Female	STI screening	Non-Reactive	Non-Reactive	Negative	Non-Reactive				Negative (not a case)
Male	STI screening	**Reactive**	Non-Reactive	**Positive**	**Reactive**	Non-Reactive	Previous Reactive		Previously treated
Female	Syphilis symptoms	Non-Reactive	Non-Reactive	Negative	Non-Reactive			Negative	Negative (not a case)
Female	STI screening	Non-Reactive	Non-Reactive	Negative	Non-Reactive				Negative (not a case)
Male	STI screening	Non-Reactive	Non-Reactive	Negative	Non-Reactive				Negative (not a case)
Male	Syphilis symptoms	**Reactive**	**Reactive**	**Positive**	**Reactive**	**1:8**		Negative	Previously treated
Male	Syphilis symptoms	Non-Reactive	Non-Reactive	Negative	Non-Reactive				Negative (not a case)
Male	STI screening	Non-Reactive	Non-Reactive	Negative	Non-Reactive				Negative (not a case)
Male	STI screening	**Reactive**	Non-Reactive	**Positive**	**Reactive**	Non-Reactive	Previous Reactive		Previously treated
Male	STI screening	Non-Reactive	Non-Reactive	Negative	Non-Reactive				Negative (not a case)
Male	STI screening	**Reactive**	**Reactive**	**Positive**	**Reactive**	**1:2**	Previous Reactive		Previously treated
Male	Syphilis symptoms	Non-Reactive	Non-Reactive	Negative	**Reactive**	Non-Reactive	Non-Reactive		**False positive**
Male	STI screening	Non-Reactive	Non-Reactive	Negative	Non-Reactive				Negative (not a case)
Male	STI screening	**Reactive**	Non-Reactive	**Positive**	**Reactive**	Non-Reactive	**Reactive**		Previously treated
Female	STI screening	Non-Reactive	Non-Reactive	Negative	Non-Reactive				Negative (not a case)
Male	STI screening	**Reactive**	Non-Reactive	**Positive**	**Reactive**	Non-Reactive	Previous Reactive		Previously treated
Male	Syphilis contact	**Reactive**	**Reactive**	**Positive**	**Reactive**	**1:4**	Previous Reactive		**New infection**
Female	STI screening	Non-Reactive	Non-Reactive	Negative	Non-Reactive				Negative (not a case)
Male	STI screening	Non-Reactive	Non-Reactive	Negative	Non-Reactive				Negative (not a case)
Male	STI screening	Non-Reactive	Non-Reactive	Negative	Non-Reactive				Negative (not a case)
Female	STI screening	Non-Reactive	Non-Reactive	Negative	Non-Reactive				Negative (not a case)
Female	STI screening	Non-Reactive	Non-Reactive	Negative	Non-Reactive				Negative (not a case)
Male	STI screening	Non-Reactive	Non-Reactive	Negative	Non-Reactive				Negative (not a case)
Female	STI screening	Non-Reactive	Non-Reactive	Negative	Non-Reactive				Negative (not a case)
Male	STI screening	Non-Reactive	Non-Reactive	Negative	Non-Reactive				Negative (not a case)
Male	STI screening	Non-Reactive	Non-Reactive	Negative	Non-Reactive				Negative (not a case)
Male	STI screening	Non-Reactive	Non-Reactive	Negative	Non-Reactive				Negative (not a case)
Male	Syphilis contact	Non-Reactive	Non-Reactive	Negative	Non-Reactive				Negative (not a case)
Male	STI screening	Non-Reactive	Non-Reactive	Negative	Non-Reactive				Negative (not a case)
Female	STI screening	Non-Reactive	Non-Reactive	Negative	Non-Reactive				Negative (not a case)
Male	STI screening	Non-Reactive	Non-Reactive	Negative	Non-Reactive				Negative (not a case)
Male	STI screening	Non-Reactive	Non-Reactive	Negative	Non-Reactive				Negative (not a case)
Male	Syphilis symptoms	Non-Reactive	Non-Reactive	Negative	Non-Reactive				Negative (not a case)
Male	Syphilis symptoms	Non-Reactive	Non-Reactive	Negative	Non-Reactive			Negative	Negative (not a case)
Female	STI screening	Non-Reactive	Non-Reactive	Negative	Non-Reactive				Negative (not a case)

Male	STI screening	Non-Reactive	Non-Reactive	Negative	Non-Reactive			Negative (not a case)
Male	Syphilis symptoms	Non-Reactive	Non-Reactive	Negative	Non-Reactive			Negative (not a case)
Female	STI screening	Non-Reactive	Non-Reactive	Negative	Non-Reactive			Negative (not a case)
Male	STI screening	Non-Reactive	Non-Reactive	Negative	Non-Reactive			Negative (not a case)
Male	Syphilis symptoms	**Reactive**	**Reactive**	**Positive**	**Reactive**	**1:2**		Previously treated
